# Inhibition of GlcNAc-Processing Glycosidases by C-6-Azido-NAG-Thiazoline and Its Derivatives

**DOI:** 10.3390/molecules19033471

**Published:** 2014-03-20

**Authors:** Jana Krejzová, Petr Šimon, Lubica Kalachova, Natallia Kulik, Pavla Bojarová, Petr Marhol, Helena Pelantová, Josef Cvačka, Rüdiger Ettrich, Kristýna Slámová, Vladimír Křen

**Affiliations:** 1 Institute of Microbiology, Academy of Sciences of the Czech Republic, Vídeňská 1083, 14220 Praha 4, Czech Republic; E-Mails: hoficek@centrum.cz (J.K.); shimon@seznam.cz (P.Š.); kalachova@biomed.cas.cz (L.K.); fialovap@email.cz (P.B.); rtepm@seznam.cz (P.M.); pelantova@biomed.cas.cz (H.P.); kren@biomed.cas.cz (V.K.); 2 Department of Biochemistry and Microbiology, Institute of Chemical Technology Prague, Technická 5, 16628 Praha 6, Czech Republic; 3 Institute of Organic Chemistry and Biochemistry, Academy of Sciences of the Czech Republic, Flemingovo nám. 2, 16610 Praha 6, Czech Republic; 4 Department of Structure and Function of Proteins, Institute of Nanobiology and Structural Biology of GCRC, Academy of Sciences of the Czech Republic, Zámek 136, 37333 Nové Hrady, Czech Republic; E-Mails: kulik@nh.cas.cz (N.K.); ettrich@nh.cas.cz (R.E.); 5 Institute of Organic Chemistry and Biochemistry, Academy of Sciences of the Czech Republic, Flemingovo nám. 2, 16610 Praha 6, Czech Republic; E-Mail: cvacka@uochb.cas.cz

**Keywords:** NAG-thiazoline, enzyme inhibition, *O*-GlcNAcase, click chemistry, azide, β-*N*-acetylhexosaminidase

## Abstract

NAG-thiazoline is a strong competitive inhibitor of GH20 β-*N*-acetyl- hexosaminidases and GH84 β-*N*-acetylglucosaminidases. Here, we focused on the design, synthesis and inhibition potency of a series of new derivatives of NAG-thiazoline modified at the C-6 position. Dimerization of NAG-thiazoline via C-6 attached triazole linkers prepared by click chemistry was employed to make use of multivalency in the inhibition. Novel compounds were tested as potential inhibitors of β-*N*-acetylhexosaminidases from *Talaromyces flavus*, *Streptomyces plicatus* (both GH20) and β-*N*-acetylglucosaminidases from *Bacteroides thetaiotaomicron* and humans (both GH84). From the set of newly prepared NAG-thiazoline derivatives, only C-6-azido-NAG-thiazoline displayed inhibition activity towards these enzymes; C-6 triazole-substituted NAG-thiazolines lacked inhibition activity against the enzymes used. Docking of C-6-azido-NAG-thiazoline into the active site of the tested enzymes was performed. Moreover, a stability study with GlcNAc-thiazoline confirmed its decomposition at pH < 6 yielding 2-acetamido-2-deoxy-1-thio-α/β-D-glucopyranoses, which presumably dimerize oxidatively into S-S linked dimers; decomposition products of NAG-thiazoline are void of inhibitory activity.

## 1. Introduction

NAG-thiazoline (2'-methyl-2-acetamido-2-deoxy-α-d-glucopyranosyl-[2,1-*d*]-∆2'-thiazoline, **1**) [1] is generally recognized as a strong competitive inhibitor of GH20 β-*N*-acetylhexosaminidases and GH84 β-*N*-acetylglucosaminidases (*O*-GlcNAcases) [2] based on mimicking of the oxazoline transition state during the hydrolytic cleavage of *N*-acetylglucosamine (GlcNAc) moiety from various substrates. Small molecule inhibitors of β-*N*-acetylhexosaminidases are very helpful tools in the studies of their physiological effects *in vivo*. However, only in case they are highly selective for just one of these enzyme groups, false observations caused by undesired inhibition of all functionally related enzymes may be avoided. Understanding the mechanism and localization of these structurally and functionally related glycosidases can help in diagnosis and treatment of serious neurodegenerative disorders such as Alzheimer’s, Tay-Sachs’ or Sandhoff’s diseases [3,4].

A kinetic study with NAG-thiazoline and a series of its derivatives with extended *N*-acyl moieties demonstrated that both types of human β-*N*-acetylhexosaminidases (GH20 and 84) are inhibited by thiazoline **1** in a very similar way (K_I_ 70 nM), while the *N*-acyl-modified analogues of **1** are generally weaker inhibitors displaying high selectivity towards *O*-GlcNAcase [5]. Several more series of various derivatives based on the NAG-thiazoline scaffold have been introduced, e.g., a set of NAG-thiazolines bearing substituents of the thiazoline ring [6], C-1 homologated thiazolines [7], and a series of C-6-acylated analogues of NAG-thiazoline [8]; however, none of those derivatives ever reached the inhibition potency of the parent compound **1**. Detailed kinetic and structural studies of *O*-GlcNAcases have enabled the design of the so far most potent and selective NAG-thiazoline-based inhibitor of human *O*-GlcNAcase called thiamet-G, which displays a 37,000-fold higher selectivity for *O*-GlcNAcase (K_I_ 21 nM) over lysosomal β-*N*-acetylhexosaminidase (K_I_ 750 μM). This inhibitor has proved its selective effect *in vivo* and is currently tested as a potential Alzheimer’s therapeutics blocking pathogenic hyperphosphorylation of protein tau [3,9].

In the present work we focused on the design, synthesis and inhibition potency of a series of new derivatives of NAG-thiazoline (**1**) modified at the C-6 position of the pyranose ring first with the azido-group (**2**), which was consequently transformed into substituted triazole-derivatives using click chemistry (**3**–**8**). Moreover, two triazole-derivatives were dimers, aiming at a possible multivalency effect in enzyme inhibition (**9**,**10**). Two GH20 β-*N*-acetylhexosaminidases and two GH84 β-*N*-acetylglucosaminidases were used as representatives of these glycosidase families in inhibition assays with the prepared NAG-thiazoline derivatives. The results of the inhibition experiments were correlated with molecular dynamics simulations of the substrate and the new inhibitor C-6-azido-NAG-thiazoline (**2**) docked into crystal structures and homology models of enzymes used in the inhibition tests.

## 2. Results and Discussion

### 2.1. Synthesis of NAG-Thiazoline Derivatives **2**–**10**

**Scheme 1:**
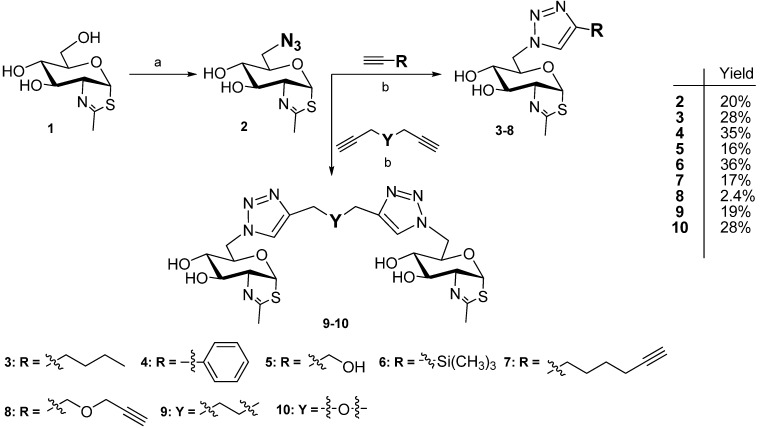
Synthetic pathway.

NAG-thiazoline (**1**), prepared from 2-acetamido-2-deoxy-1,3,4,6-tetraacetyl-β-d-glucopyranoside using Lawesson’s reagent followed by Zemplén deacetylation [1], was used as starting material for derivatizations. Compound **1** was activated at C-6 by tosylation with TsCl in pyridine followed by the reaction with NaN_3_ in DMF [10] and the obtained azido derivative **2** was then treated with respective alkyne derivatives under Cu^+^ catalysis (Cu-AAC, copper catalyzed azide-alkyne cycloaddition). Eight new derivatives of NAG-thiazoline attached *via* triazole ring at C-6 were prepared. Two structures of these series are dimeric NAG-thiazolines bridged by aliphatic or etheric linkers (Scheme 1), which are non-hydrolysable *in vivo*.

*Reagents and Conditions*: (**a**) TsCl, pyridine, 20 °C, 1 h; then NaN_3_, DMF, 60 °C, 16 h; (**b**) CH_3_CN, DIPEA, CuI, 20 °C, 16 h.

Excess of monofunctional alkyne afforded triazole derivatives substituted by a sugar unit at N-1 of the triazole moiety (Scheme 1). Half equivalent of the bifunctional alkyne linked two NAG-thiazoline moieties in an analogous way. The influence of the respective linkers attached to the triazole moiety was evaluated to select the optimal linker type for multivalent presentation of this inhibitor, finally, aliphatic (octa-1,7-diyne) and etheric (dipropargylether) dialkynes were employed. Reaction conditions of Cu-AAC were improved with respect to the present acido-labile thiazoline moiety; the optimized reactions catalyzed by CuI in acetonitrile and DIPEA afforded good yields. 

### 2.2. Stability of the NAG-Thiazoline Parent Structure **1**

Since some GlcNAc-processing enzymes such as fungal β-*N*-acetylhexosaminidases optimally act (and are usually investigated) under acidic conditions [11] we examined the fate of NAG-thiazoline at pH below 7 at 35 °C. As a result, the acid-labile thiazoline ring quickly (within minutes) decomposed at pH < 6; at pH 6 the stability was safely acceptable for experiments that last maximally up to one hour. A thorough HPLC monitoring of NAG-thiazoline stability at pH 6.5 revealed a quasi-linear decomposition rate of ca 1% per hour, decreasing to 0.5% per hour after 3 h. After 24 h, the content of intact NAG-thiazoline decreased to ca 70% of the original concentration. This demonstrates that the use of NAG-thiazoline for long term experiments (in the range of days) at pH < 7 is unadvisable since the decomposition rate is not negligible under these conditions; at lower pH the decomposition is expected to proceed much faster.

**Scheme 2:**
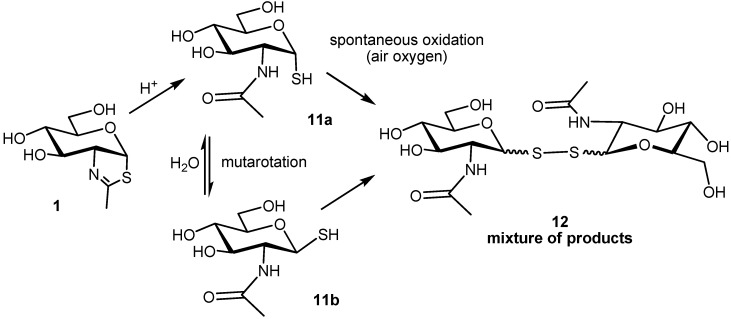
Decomposition products of NAG-thiazoline **1**; 2-acetamido-2-deoxy-1-thio-α-d-glucopyranose (**11a**), its β-anomer **11b** formed by mutarotation and a mixture of oxidation products (tentative structure) spontaneously originating by the action of air oxygen.

The direct decomposition product of NAG-thiazoline is 2-acetamido-2-deoxy-1-thio-α-d-glucopyranose (**11a**). Besides expected mutarotation to its β-anomer **11b**, both these compounds rapidly undergo spontaneous oxidation by air oxygen to form a rich mixture of products, presumably of GlcNAc-S-(1↔1)-S-GlcNAc type (*m/z* = 456) (Scheme 2). Importantly, neither the hydrolytic products **11a** and **11b** nor the originating oxidation products displayed any inhibition effects comparable to thiazoline **1** (as shown in *Talaromyces flavus* β-*N*-acetylhexosaminidase). After application of dithiothreitol, the oxidation mixture was reduced back to **11a** and **11b**, as confirmed by *in situ* NMR and HPLC experiments.

### 2.3. Inhibiton of GH20 and GH84 Glycosidases with NAG-Thiazoline Derivatives

The parent compound NAG-thiazoline (**1**) is a well established competitive mechanism-based inhibitor of the GH20 β-*N*-acetylhexosaminidases and GH84 β-*N*-acetylglucosaminidases [1,5]. Based on its structure, a number of analogues were designed and tested as β-*N*-acetylhexosaminidase inhibitors, including various *N*-acyl-derivatives [3,5,6] and the C-6-acylated derivatives [8]. Thus, the prepared monomeric (**2**–**8**) and dimeric (**9**,**10**) derivatives of NAG-thiazoline bearing the azido or triazole moiety at C-6 were designed as potential competitive inhibitors of GlcNAc-processing enzymes from the families 20 and 84 of glycoside hydrolases.

One bacterial and one eukaryotic model enzyme of each glycosidase family were selected for the inhibition study: the GH20 β-*N*-acetylhexosaminidases from *Streptomyces plicatus* (bacterial) and *Talaromyces flavus* (fungal) and GH84 β-*N*-acetylglucosaminidases from *Bacteroides thetaiotaomicron* (bacterial) and humans. Bacterial and human enzymes were prepared by heterologous expression in *E. coli* using standard procedures and purified by metal affinity chromatography from the lysed cells. β-*N*-Acetylhexosaminidase from *T. flavus* was expressed extracellularly in *Pichia pastoris* and purified by cation exchange chromatography from the culture media. All new compounds were tested with all of the enzymes but only the C-6-azido-NAG-thiazoline (**2**) displayed inhibition activity towards these enzymes; the triazole-substituted NAG-thiazolines lacked any inhibition activity against the enzymes used. The results of the Michaelis-Menten kinetic experiments with NAG-thiazoline (**1**) and C-6-azido-NAG-thiazoline (**2**) using *p*NP-β-GlcNAc as a substrate are summarized in Table 1.

**Table 1:** Inhibition of β-*N*-acetylhexosaminidases and β-*N*-acetylglucosaminidases by NAG-thiazoline **1** and its C-6-azido derivative **2** (substrate *p*NP-β-GlcNAc; pH 7; 25 °C).

		K_I_ [µM]	K_I_ [µM]
Enzyme		NAG-thiazoline (1)	C-6-azido-NAG-thiazoline (2)
**β-*N*-acetylhexosaminidase**	*T. flavus*	42.7 ± 1.9	211.9 ± 16.3
	*S. plicatus*	24 ± 5	n.d.
***O*-GlcNAcase**	*B. thetaiotaomicron*	0.029 ± 0.003	3.1 ± 0.3
	human	0.18 ± 0.03	19.3 ± 11.9

n.d. = not determined.

The inhibition constants presented in Table 1 clearly indicate the stronger effect of both inhibitors with the GH84 β-*N*-acetylglucosaminidases, while with GH20 β-*N*-acetylhexosaminidases the inhibition activity of the tested inhibitors is significantly (up to three orders of magnitude) lower or even negligible as in the case of bacterial β-*N*-acetylhexosaminidase and the azido-derivative **2**. However, in all cases the newly designed C-6-azido-NAG-thiazoline (**2**) performed somewhat worse than the original NAG-thiazoline; with *O*-GlcNAcases the inhibition constants appeared approximately hundred times higher. We can conclude that both tested inhibitors are highly selective for the GH84 glycosidases while the C-6-azido derivative **2** remains less effective than the parent compound **1**. Similar result has been shown previously with other structurally modified NAG-thiazolines [5,6,8] with the only exception of the potential Alzheimer’s therapeutic thiamet-G [3]. Thus, new pathways are still to be found that lead to derivatives conquering the inhibition potency of **1**.

### 2.4. Interactions and Calculated Binding Energies of C-6-azido-NAG-thiazoline (**2**) in The Active Site of Enzymes

**Figure 1:**
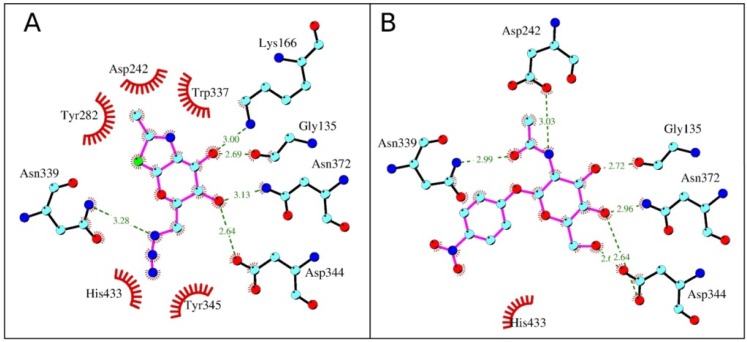
C-6-Azido-NAG-thiazoline (**2**) (**A**) and substrate *p*NP-β-GlcNAc (**B**) in the active site of bacterial β-*N*-acetylglucosaminidase (*B. thetaiotaomicron*). Color scheme: carbon atom–cyan, oxygen–red, nitrogen–blue, sulfur–green, hydrogens are omitted; hydrogen bonds–green dashed line with marked donor-acceptor length in Ångstroms; residues participating in π-cation interaction are shown schematically.

To obtain a deeper understanding of the inhibition on a molecular scale, we performed molecular docking and consequent molecular dynamics simulations of inhibitors **1** and **2** and the artificial substrate *p*NP-β-GlcNAc. In molecular dynamics simulations the solvent is explicitly included, and after initial equilibration at the given temperature the full conformational space of the substrate/inhibitor-enzyme complexes is sampled, which significantly improves further analysis of the initial docked poses and gives a more realistic understanding of the substrate-enzyme interaction. In the simulations substrate/inhibitor-enzyme complexes adapted to the environment and reached equilibrium around 5 ns as estimated by root mean square deviation, and only the production run hereafter was used for analysis. For energy calculations complex conformations from the last 2 ns of the production runs were used to ensure that they represent the equilibrated substrate/inhibitor-enzyme complex. The binding energy hereby is estimated as the sum of the intermolecular energy calculated by the force field and the torsional free-energy penalty. Hereby, the intermolecular energy includes terms for electrostatic energy, hydrogen bonding, van der Waals interactions, and a desolvation energy estimate. A comparison of the calculated binding energies and type of interactions for the various complexes is presented in Table 2. The strongest binding was observed in the complex of the bacterial *O*-GlcNAcase and C-6-azido-NAG-thiazoline (**2**), whose binding energy is significantly lower than the one calculated for the substrate and also inhibitor **1**. The interaction of the inhibitor with the active site of bacterial *O*-GlcNAcase is enhanced by two π-cation interactions, one by the azido-group at the C-6 atom with residues Tyr345 and His433, the other one by the positively charged nitrogen atom in the oxazolinium ring involving Tyr282 and Trp337 compared to *p*NP-β-GlcNAc (Figure 1). However, His433 is not conserved throughout the family and in human β-*N*-acetylglucosaminidase this position is filled by the smaller and neutral valine in the vicinity of the inhibitor’s azido-group.

**Table 2:** Binding energies * in the molecular dynamics simulations calculated by AutoDock (number of hydrogen bonds throughout the simulations).

Enzyme		NAG-thiazoline (1) [kcal/mol]	C-6-azido-NAG-thiazoline (2) [kcal/mol]	*p*NP-β-GlcNAc [kcal/mol]
**β-*N*-acetylhexosaminidase**	*S. plicatus*	−9.22 (6−7)	−7.36 (3−5)	−9.48 (5−8)
	*T. flavus*	−7.63 (3−6)	−7.71 (3−4)	−10.19 (6−9)
***O*-GlcNAcase **	*B. thetaiotaomicron*	−7.14 (4−5)	−8.65 (4−5)	−7.75 (6−7)
	human	−6.42 (3−4)	−7.16 (3−4)	−7.45 (5−6)

* Lower binding energy corresponds to better binding.

**Figure 2:**
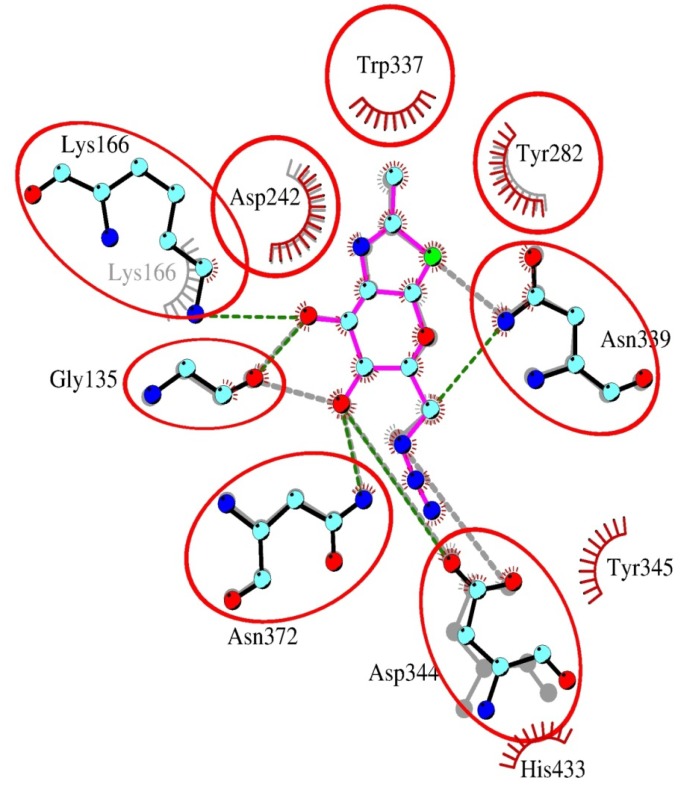
Overlay of NAG-thiazoline (**1**) and C-6-azido-NAG-thiazoline (**2**) and in the active site of bacterial β-*N*-acetylglucosaminidase. Bacterial residues and C-6-azido-NAG-thiazoline are labeled in vivid colors using the color scheme analogous to Figure 1. Bacterial residues and NAG-thiazoline are shown in grey. Common residues participating in hydrogen bond or π-cation interactions are in circles.

**Figure 3:**
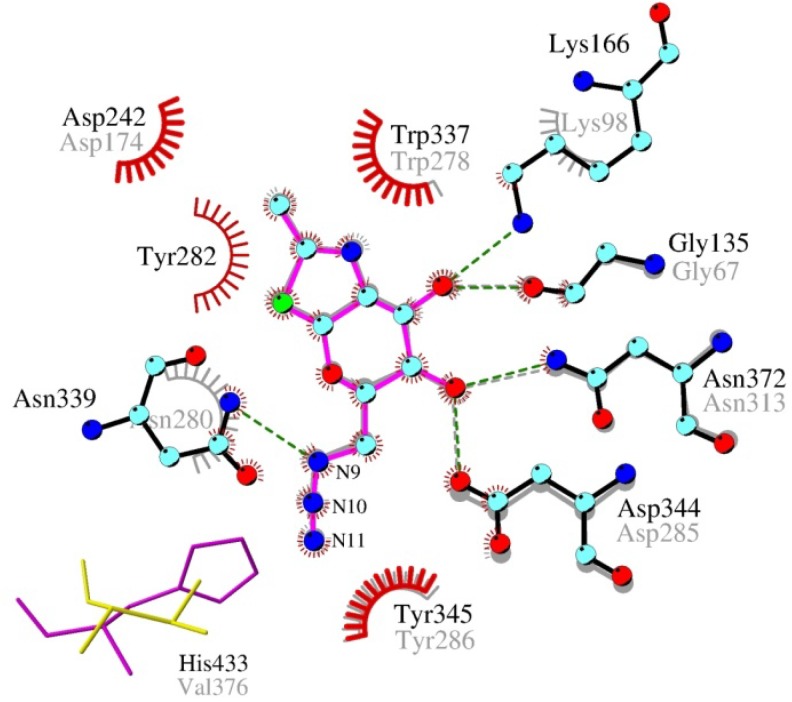
Overlay of C-6-azido-NAG-thiazoline (**2**) in the active site of human and bacterial β-*N*-acetylglucosaminidases. Bacterial residues are labeled in the upper lines, human Lys98 and Asn280 are shown schematically only, as they are not in hydrogen bond distance to the inhibitor. Bacterial active site amino acid residues are shown in color, human enzyme is presented in grey. Residues His433 (magenta) in the bacterial enzyme and the corresponding Val376 (yellow) in the human enzyme are shown in stick representation. Val376 is unable to form a π-cation interaction with the azido-group (N9-N10-N11) of **2**. Other interactions are similar to the bacterial enzyme. Color scheme is the same as in Figure 1.

Surprisingly, inhibitor **2** appeared to bind better than **1** into the active site of both bacterial and human β-*N*-acetylglucosaminidases. This improvement could be explained by π-cation interaction of C-6 azido-group with aromatic residues (Figure 2). In the case of human GH84 enzyme this interaction is weaker and occurs just with Tyr286 (corresponds to Tyr345 in bacterial GH84). This together with the loss of a hydrogen bonding interaction of human Asn280 with C-6-azido-NAG-thiazoline (**2**) caused the significantly weaker binding of inhibitor **2** (Figure 3) to human β-*N*-acetylglucosaminidase.

**Figure 4:**
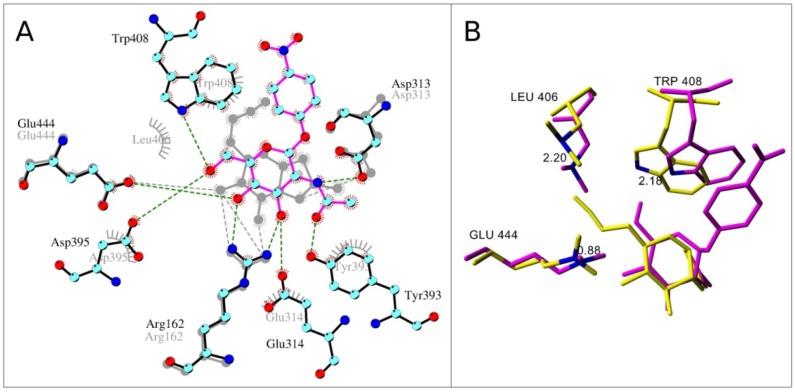
Overlay of *p*NP-β-GlcNAc and C-6-azido-NAG-thiazoline (**2**) docked into the active site of bacterial β-*N*-acetylhexosaminidase (*S. plicatus*). (**A**) Hydrogen bond interaction in the active site. Color scheme is like in Figure 1. Enzyme with docked *p*NP-β-GlcNAc is shown in color and C-6-azido-NAG-thiazoline in grey. (**B**) Active site amino acids changed orientation during MD with C-6-azido-NAG-thiazoline (yellow color). Enzyme and *p*NP-β-GlcNAc are shown in magenta. Distances between corresponding atoms (Å; in blue) of identical residues are labeled.

**Figure 5:**
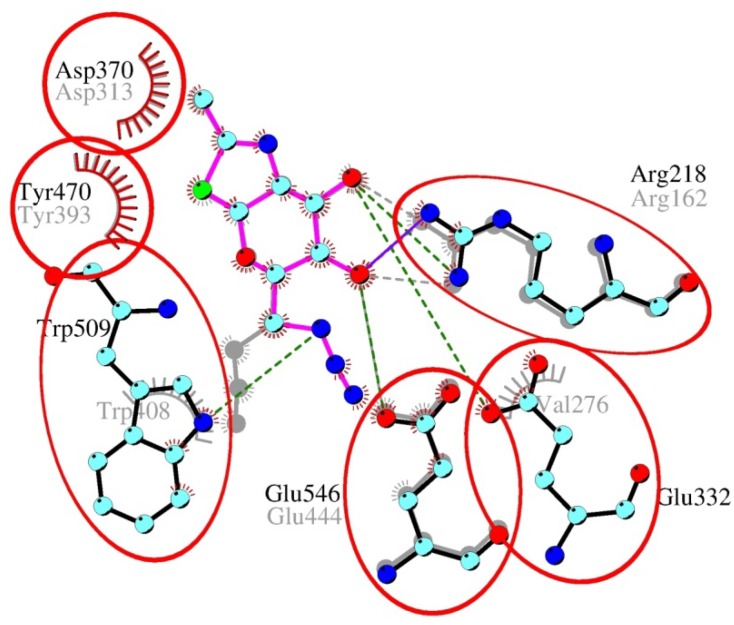
Overlay of C-6-azido-NAG-thiazoline (**2**) in the active site of β-*N*-acetylhexosaminidases from *T. flavus* (in color, active site amino acids are labeled in the upper line) and *S. plicatus* (grey, active site amino acids are labeled in the lower line). Common amino acid residues participating in the interaction are emphasized by circles. Trp408 from *S. plicatus* contributes only to the hydrophobic interaction with the inhibitor. Due to the slightly different orientation of the inhibitor in the active site of both enzymes, Arg162 from *S. plicatus* contributes to hydrogen bonds with atoms O-3 and O-4, while Arg218 (from the *T. flavus* enzyme) is able to form just one hydrogen bond with the O-3 atom.

A different character of interactions was observed in the case of GH20 β-*N*-acetylhexosaminidases. Here, the azido-group of inhibitor **2** docked into the active site is not surrounded by aromatic residues like in bacterial *O*-GlcNAcase, weakening the stability of the resulting complexes. Generally, the affinity of β-*N*-acetylhexosaminidases to modified NAG-thiazoline **2** is lower than to the substrate that has more hydrogen bonding partners and a π-π stacking interaction of the *p*-nitrophenyl group with tryptophan residues (Figure 4A). In the fungal enzyme, the C-3 hydroxyl group of **2** contributes to one additional hydrogen bond with Glu322 and Trp509. Again, residue Glu322 is not conserved throughout the family and in the bacterial β-*N*-acetylhexosaminidase a valine is present in this position. Equilibrium orientation of the azido-group in C-6 modified NAG-thiazoline in the active site of bacterial GH20 is different from NAG-thiazoline and substrate, because orientation of some residues in the vicinity of C-6 atom is changed to accommodate the azido-group (Figure 4B), which explains the minor potential of compound **2** to inhibit this bacterial enzyme. In the case of *T. flavus* enzyme, the equilibrium orientations of inhibitor **2** and substrate were similar and did not initiate distortions in the vicinity of the C-6 atom (Figure 5).

Overall, the results of molecular docking appear to be in a good correlation with the performed kinetic experiments, predicting the best inhibition for bacterial *O*-GlcNAcase and suggesting a molecular explanation for the undetermined inhibition constant with bacterial β-*N*-acetylhexosaminidase. These results give us an idea about potency of the mentioned enzymes to tolerate modifications for future inhibitor constructions.

## 3. Experimental

### 3.1. General Methods

All chemicals were purchased from Sigma-Aldrich (Prague, Czech Republic). Reactions were monitored by TLC with pre-coated silica gel 60 F_254_ aluminum sheets from Merck (Prague, Czech Republic), detected with UV light and/or charred with sulfuric acid (5% H_2_SO_4_ in EtOH). Compounds were purified by column flash chromatography with silica gel 60 (230–240 mesh, Merck). Solvents were distilled and dried according to the standard procedures before use.

### 3.2. NMR Spectroscopy

NMR spectra were recorded on a Bruker Avance III 400 MHz spectrometer (400.00 MHz for ^1^H, 100.58 MHz for ^13^C at 30 °C in CD_3_OD-compounds **2**–**10**) and a Bruker Avance III 700 MHz spectrometer (700.13 MHz for ^1^H, 176.07 MHz for ^13^C at 30 °C in D_2_O-compounds **11a** and **11b**). Residual signals of solvent were used as internal standards (δ_H_ 3.330 ppm, δ_C_ 49.30 ppm for CD_3_OD; δ_H_ 4.508 ppm for D_2_O). Carbon chemical shifts in D_2_O were referenced to acetone (δ_C_ 30.50 ppm). NMR experiments ^1^H-NMR, ^13^C-NMR, gCOSY, gHSQC, and gHMBC were performed using the manufacturer’s software. ^1^H-NMR and ^13^C-NMR spectra were zero filled to fourfold data points and multiplied by window function before Fourier transformation. Two-parameter double-exponential Lorentz-Gauss function was applied for ^1^H to improve resolution and line broadening (1 Hz) was applied to get better ^13^C signal-to-noise ratio. Chemical shifts are given in δ-scale with digital resolution justifying the reported values to three (δ_H_) or two (δ_C_) decimal places. The full NMR spectra of the newly prepared compounds **2**–**10** are presented in the Supplementary material.

Proton spin systems of thiazoline and triazole-linker moieties were assigned by COSY and by HSQC transferred to carbons; HMBC experiment enabled to join the above mentioned partial structures together. Thiazoline structure was proved by the presence of methyl doublet (*J* = 2.0 or 2.1 Hz) correlated in HMBC to carbons C-1 and C-2. Dimer formation (compounds **9** and **10**) was unambiguously confirmed by the auto-correlation cross peak of the central carbon of the linker (C-3' for **10** and C-5' for **9**). ^1^H and ^13^C NMR spectra of compounds **2**–**10** are provided in the Supplementary information.

### 3.3. Mass Spectrometry

The exact masses were measured using LTQ Orbitrap XL hybrid mass spectrometer (Thermo Fisher Scientific, Waltham, MA, USA) equipped with an electrospray ion source. The mobile phase consisted of methanol/water (4:1), flow rate 30 μL/min, and the samples were injected using a 2-μL loop. The mass spectra of positively charged ions were internally calibrated using protonated phthalic anhydride as lock mass. Data were acquired and processed using Xcalibur software (Thermo Fisher Scientific). HRMS spectra of compounds **2**–**10** are provided in the Supplementary information.

### 3.4. Synthesis of NAG-Thiazoline Derivatives **2**–**10**

*6-Azido-1,2-dideoxy-2'-methyl-α-d-glucopyrano-[2,1-d]-*∆2'-*thiazoline* (**2**): 1,2-Dideoxy-2'-methyl-α-d-glucopyrano-[2,1-*d*]-∆2'-thiazoline prepared according to Knapp and coworkers [1] (**1**, 1 g 4.56 mmol) was dissolved in the mixture of dry pyridine (10 mL) and dry CH_2_Cl_2_ (10 mL), cooled to 0 °C and TsCl (1 g, 5.25 mmol) was added. Reaction mixture was stirred 1 h at room temperature and then CH_2_Cl_2_ (10 mL) was added. Organic phase was washed twice with H_2_O and dried over Na_2_SO_4_. Solvents were evaporated, the tosylated derivative was dried *in vacuo*, dissolved in dry DMF (15 mL) and NaN_3_ (800 mg, 11.9 mmol, 2.6 eq) was added. Reaction mixture was stirred at 60 °C overnight. Title compound **2** was at first purified by column chromatography (silica gel, EtOAc/MeOH = 9:1). Isolated mixture of expected product **2** and corresponding 3,6-anhydro-derivative were separated by gel filtration using Sephadex LH-20 (GE Healthcare by AP Czech, Prague, Czech Republic) using 80% MeOH as an eluent. Product **2** was isolated as a white solid (223 mg, 20%). ^1^H-NMR (400.00 MHz, CD_3_OD): 2.288 (3H, d, *J* = 2.1 Hz, CH_3_), 3.418 (2H, m, H-6), 3.471 (1H, m, H-5), 3.588 (1H, ddd, *J* = 8.8, 3.6, 1.0 Hz, H-4) 4.169 (1H, ddd, *J* = 4.7, 3.6, 0.7 Hz, H-3), 4.377 (1H, dddq, *J* = 7.0, 4.7, 1.0, 2.1 Hz, H-2), 6.394 (1H, d, *J* = 7.0 Hz, H-1). ^13^C-NMR (100.58 MHz, CD_3_OD): 20.96 (CH_3_), 53.62 (C-6), 72.67 (C-4), 74.35 (C-3), 75.24 (C-5), 80.83 (C-2), 90.89 (C-1), 170.96 (=C-). HRMS: C_8_H_12_O_3_N_4_NaS calcd. 267.05223; *m/z* [M+Na]^+^ found 267.05227.

*6-(4-Butyltriazolyl)-1,2-dideoxy-2'-methyl-α-d-glucopyrano-[2,1-d]-*∆2'-*thiazoline* (**3**): Compound **2** (100 mg, 0.41 mmol) was dissolved in the mixture of acetonitrile (3 mL) and *N,N*-diisopropylethylamine (0.2 mL). Hexyne (0.1 mL) and CuI (30 mg) were added and reaction mixture was stirred overnight at room temperature. The product was isolated after evaporation of solvents by column chromatography (silica gel, EtOAc-MeOH = 9:1). Butyl derivative **3** was isolated as a white solid (38 mg, 28%). ^1^H-NMR (400.00 MHz, CD_3_OD): 0.967 (3H, t, *J* = 7.4 Hz, H-6'), 1.395 (2H, m, H-5'), 1.665 (2H, m, H-4'), 2.237 (3H, d, *J* = 2.1 Hz, CH_3_), 2,710 (2H, dt, *J* = 0.6, 7.5 Hz, H-3'), 3.575 (2H, m, H-4, H-5), 4.245 (1H, dd, Σ*J* = 6.8 Hz, H-3), 4.380 (1H, dddq, Σ*J* = 18.1 Hz, H-2), 4.474 (1H, dd, *J* = 14.4, 8.0 Hz, H-6u), 4.717 (1H, dd, *J* = 14.4, 1.9 Hz, H-6d), 6.330 (1H, d, *J* = 7.0 Hz, H-1), 7.695 (1H, t, *J* = 0.6 Hz, H-1'). ^13^C-NMR (100.58 MHz, CD_3_OD): 14.41 (C-6'), 20.82 (CH_3_), 23.39 (C-5'), 26.18 (C-3'), 32.96 (C-4'), 53.25 (C-6), 73.05 (C-4), 74.17 (C-3), 74.50 (C-5), 80.90 (C-2), 90.66 (C-1), 124.41 (C-1'), 149.36 (C-2'), 170.84 (=C-). HRMS: C_14_H_23_O_3_N_4_S calcd. 327.14854; *m/z* [M+H]^+^ found 327.14848.

*1,2-Dideoxy-2'-methyl-6-(4-phenyltriazolyl)-α-d-glucopyrano-[2,1-d]-*∆2'-*thiazoline* (**4**): Compound **4** was prepared analogously as described for the compound **3** from azide **2** (100 mg) and phenylacetylene (0.1 mL) yielding title compound **4** as a white solid (50 mg, 35%). ^1^H-NMR (400.00 MHz, CD_3_OD): 2.239 (3H, d, *J* = 2.1 Hz, CH_3_), 3.631 (1H, ddd, *J* = 9.1, 3.2, 1.0 Hz, H-4), 3.689 (1H, dddd, *J* = 9.1, 7.7, 2.5, 0.4 Hz, H-5), 4.269 (1H, dd, *J* = 4.7, 3.2 Hz, H-3), 4.399 (1H, dddq, *J* = 7.0, 4.7, 1.0, 2.1 Hz, H-2), 4.598 (1H, dd, *J* = 14.4, 7.7 Hz, H-6u), 4.806 (1H, dd, *J* = 14.4, 2.5 Hz, H-6d), 6.368 (1H, d, *J* = 7.0 Hz, H-1), 7.352 (1H, m, H-*para*), 7.443 (2H, m, H-*meta*), 7.825 (2H, m, H-*ortho*), 8.283 (1H, s, H-1'). ^13^C-NMR (100.58 MHz, CD_3_OD): 20.92 (CH_3_), 53.38 (C-6), 72.29 (C-4), 74.18 (C-3), 74.39 (C-5), 80.74 (C-2), 90.63 (C-1), 123.60 (C-1'), 126.95 (C-*ortho*), 129.59 (C-*para*), 130.23 (C-*meta*), 131.92 (C-*ipso*), 148.94 (C-2'), 171.10 (=C-). HRMS: C_16_H_19_O_3_N_4_S calcd. 347.11724; *m/z* [M+H]^+^ found 347.11704.

*1,2-Dideoxy-6-(4-hydroxymethyltriazolyl)-2'-methyl-α-d-glucopyrano-[2,1-d]-*∆2'-*thiazoline* (**5**): Compound **5** was prepared as described for the compound **3** from azide **2** (100 mg) and propargylalcohol (0.1 mL) yielding hydroxymethyl derivative **5** as a white solid (20 mg, 16%). ^1^H-NMR (400.00 MHz, CD_3_OD): 2.241 (3H, d, *J* = 2.0 Hz, CH_3_), 3.562 (1H, ddd, *J* = 9.1, 3.3, 1.0 Hz, H-4), 3.639 (1H, dddd, *J* = 9.1, 7.9, 2.5, 0.4 Hz, H-5), 4.222 (1H, dd, *J* = 4.6, 3.3 Hz, H-3), 4.372 (1H, dddq, *J* = 7.0, 4.6, 1.0, 2.0 Hz, H-2), 4.542 (1H, dd, *J* = 14.5, 7.9 Hz, H-6u), 4.696 (2H, d, *J* = 0.6 Hz, H-3'), 4.751 (1H, dd, *J* = 14.5, 2.5 Hz, H-6d), 6.337 (1H, d, *J* = 7.0 Hz, H-1), 7.887 (1H, t, *J* = 0.6 Hz, H-1'). ^13^C-NMR (100.58 MHz, CD_3_OD): 20.86 (CH_3_), 53.26 (C-6), 56.77 (C-3'), 72.89 (C-4), 74.20 (C-3), 74.39 (C-5), 80.78 (C-2), 90.70 (C-1), 124.41 (C-1'), 149.20 (C-2'), 170.95 (=C-). HRMS: C_11_H_17_O_4_N_4_S calcd. 301.09650; *m/z* [M+H]^+^ found 301.09650.

*1,2-Dideoxy-2'-methyl-6-(4-trimethylsilyltriazolyl)-α-d-glucopyrano-[2,1-d]-*∆2'-*thiazoline* (**6**): Compound **6** was prepared as described for compound **3** from azide **2** (100 mg) and trimethylsilylacetylene (0.1 mL) yielding trimethylsilyl derivative **6** as a white solid (50 mg, 36%). ^1^H-NMR (400.00 MHz, CD_3_OD): 0.334 (9H, s, Si(CH_3_)_3_), 2.261 (3H, d, *J* = 2.0 Hz, CH_3_), 3.594 (1H, ddd, *J* = 9.2, 3.1, 0.8 Hz, H-4), 3.641 (1H, ddd, *J* = 9.2, 7.6, 2.3 Hz, H-5), 4.272 (1H, dd, *J* = 4.5, 3.1 Hz, H-3), 4.405 (1H, m, H-2), 4.569 (1H, dd, *J* = 14.4, 7.6 Hz, H-6u), 4.796 (1H, dd, *J* = 14.4, 2.3 Hz, H-6d), 6.341 (1H, d, *J* = 7.0 Hz, H-1), 7.970 (1H, s, H-1'). ^13^C-NMR (100.58 MHz, CD_3_OD): −0.78 (Si(CH_3_)_3_), 21.10 (CH_3_), 52.77 (C-6), 72.99 (C-4), 74.27 (C-3), 74.58 (C-5), 80.82 (C-2), 90.61 (C-1), 132.82 (C-1'), 147.47 (C-2'), 171.39 (=C-). HRMS: C_13_H_23_O_3_N_4_SSi calcd. 343.12546; *m/z* [M+H]^+^ found 343.12537.

*1,2-Dideoxy-6-[4-(hex-5-ynyl)triazolyl]-2'-methyl-α-d-glucopyrano-[2,1-d]-*∆2'*-thiazoline* (**7**) *and 1,4-Bis[(1,2-dideoxy-2´-methyl-α-d-glucopyrano-[2,1-d]-*∆2´*-thiazolin-6-yl)-triazol-4-yl]-butane* (**9**)

Compound **2** (300 mg, 1.23 mmol) was dissolved in the mixture of acetonitrile (15 mL) and *N,N*-diisopropylethylamine (0.6 mL). Octa-1,7-diyne (0.08 mL) and CuI (150 mg) were added and the reaction mixture was stirred overnight at room temperature. The products were isolated after evaporation of solvents by column chromatography (silica gel, CHCl_3_/MeOH = 6:1). Monomeric derivative **7** was isolated as a white solid (75 mg, 17%) and dimeric derivative **9** was then eluted by methanol gradient as a white solid (69 mg, 19%).

*Compound* **7**: ^1^H-NMR (400.00 MHz, CD_3_OD): 1.571 (2H, m, H-5'), 1.802 (2H, m, H-4'), 2.233 (3H, m, H-6', H-8'), 2.242 (3H, d, *J* = 2.1 Hz, CH_3_), 2.738 (2H, dt, *J* = 0.6, 7.4 Hz, H-3'), 3.567 (2H, m, H-4, H-5), 4.249 (1H, m, H-3), 4.382 (1H, dqm, *J* = 7.0, 2.1 Hz, H-2), 4.474 (1H, m, H-6u), 4.723 (1H, m, H-6d), 6.331 (1H, d, *J* = 7.0 Hz, H-1), 7.718 (1H, t, *J* = 0.6 Hz, H-1'). ^13^C-NMR (100.58 MHz, CD_3_OD): 19.06 (C-6'), 20.81 (CH_3_), 25.95 (C-3'), 29.22 (C-5'), 29.78 (C-4'), 53.28 (C-6), 70.01 (C-8', 73.08 (C-4), 74.14 (C-3), 74.50 (C-5), 80.81 (C-2), 85.12(C-7'), 90.62 (C-1), 124.51 (C-1'), 149.01 (C-2'), 171.10 (=C-). HRMS: C_16_H_22_O_3_N_4_NaS calcd. 373.13048; *m/z* [M+Na]^+^ found 373.13033. 

*Compound* **9**: ^1^H-NMR (400.00 MHz, CD_3_OD): 1.734 (2H, m, H-4'), 2.228 (3H, d, *J* = 2.0 Hz, CH_3_), 2.754 (2H, m, H-3'), 3.561 (1H, m, H-4), 3.584 (1H, m, H-5), 4.230 (1H, m, H-3), 4.371 (1H, dddq, *J* = 7.0, 4.8, 0.8, 2.0 Hz, H-2), 4.485 (1H, m, H-6u), 4.712 (1H, m, H-6d), 6.329 (1H, d, *J* = 7.0 Hz, H-1), 7.717 (1H, t, *J* = 0.6 Hz, H-1'). ^13^C-NMR (100.58 MHz, CD_3_OD): 20.86 (CH_3_), 26.18 (C-3'), 30.00 (C-4'), 53.25 (C-6), 73.01 (C-4), 74.20 (C-3), 74.50 (C-5), 80.90 (C-2), 90.70 (C-1), 124.62 (C-1'), 149.03 (C-2'), 170.82 (=C-). HRMS: C_24_H_35_O_6_N_8_S_2_ calcd. 595.21155; *m/z* [M+H]^+^ found 595.21133.

*1,2-Dideoxy-2'-methyl-6-[4-(propargyloxymethyl)-triazolyl]-α-d-glucopyrano-[2,1-d]-*∆2'*-thiazoline* (**8**) *and bis{[(1,2-dideoxy-2´-methyl-α-d-glucopyrano-[2,1-d]-*∆2´*-thiazoline-6-yl)-triazol-4-yl]-methyl} ether* (**10**) 

Compounds **8** and **10** were prepared as described for the compounds **7** and **9**, respectively, from the azide **2** (300 mg) and dipropargylether (0.08 mL) yielding monomeric derivative **8** as a white solid (10 mg, 2.4%) and dimeric derivative **10** as a white solid (100 mg, 28%).

*Compound* **8**: ^1^H-NMR (400.00 MHz, CD_3_OD): 2.242 (3H, d, *J* = 2.1 Hz, CH_3_), 2.915 (1H, t, *J* = 2.4 Hz, H-6'), 3.568 (1H, m, H-4), 3.610 (1H, m, H-5), 4.211 (2H, d, *J* = 2.4 Hz, H-4'), 4.241 (1H, dd, *J* = 4.5, 2.7 Hz, H-3), 4.379 (1H, dddq, *J* = 7.0, 4.5, 0.9, 2.1 Hz, H-2), 4.538 (1H, dd, *J* = 14.4, 7.7 Hz, H-6u), 4.702 (2H, d, *J* = 0.5 Hz, H-3'), 4.762 (1H, dd, *J* = 14.4, 2.3 Hz, H-6d), 6.336 (1H, d, *J* = 7.0 Hz, H-1), 7.954 (1H, t, *J* = 0.5 Hz, H-1'). ^13^C-NMR (100.58 MHz, CD_3_OD): 20.83 (CH_3_), 53.33 (C-6), 58.28 (C-4'), 63.47 (C-3'), 72.99 (C-4), 74.16 (C-3), 74.35 (C-5), 76.73 (C-6'), 80.49 (C-5'), 80.83 (C-2), 90.65 (C-1), 126.62 (C-1'), 145.41 (C-2'), 170.93 (=C-). HRMS: C_14_H_19_O_4_N_4_S calcd. 339.11215; *m/z* [M+H]^+^ found 339.11203.

*Compound* **10**: ^1^H-NMR (400.00 MHz, CD_3_OD): 2.236 (6H, d, *J* = 2.1 Hz, CH_3_), 3.567 (2H, ddd, *J* = 9.1, 2.9, 1.0 Hz, H-4), 3.615 (2H, dddd, *J* = 9.1, 7.6, 2.4, 0.4 Hz, H-5), 4.238 (2H, dd, *J* = 4.5, 2.9 Hz, H-3), 4.378 (2H, dddq, *J* = 7.0, 4.5, 1.0, 2.1 Hz, H-2), 4.549 (2H, dd, *J* = 14.4, 7.6 Hz, H-6u), 4.667 (4H, d, *J* = 0.4 Hz, H-3'), 4.763 (2H, dd, *J* = 14.4, 2.4 Hz, H-6d), 6.341 (2H, d, *J* = 7.0 Hz, H-1), 7.967 (2H, t, *J* = 0.4 Hz, H-1'). ^13^C-NMR (100.58 MHz, CD_3_OD): 20.86 (CH_3_), 53.32 (C-6), 64.12 (C-3'), 72.95 (C-4), 74.15 (C-3), 74.36 (C-5), 80.81 (C-2), 90.68 (C-1), 126.62 (C-1'), 145.73 (C-2'), 170.91 (=C-). HRMS: C_22_H_31_O_7_N_8_S_2_ calcd. 583.17516; *m/z* [M+H]^+^ found 583.17503.

### 3.5. Stability of NAG-Thiazoline **1**

NAG-thiazoline (**1**; 200 mM) was incubated in 50 mM K_2_HPO_4_/KH_2_PO_4_ buffer pH 6.5 at 35 °C and 850 rpm. Aliquots (10 μL) were taken at regular time intervals (30–60 min), mixed with 95% CH_3_CN (40 μL) and analyzed by HPLC. Chromatography was carried out on the Shimadzu Prominence UFLC system (Kyoto, Japan) consisting of DGU-20A mobile phase degasser, two LC-20AD solvent delivery units, SIL-20ACHT cooling autosampler, CTO-10AS column oven and SPD-M20A diode array detector. The HILIC column TSKgel Amide-80 (250 × 4.6 mm i.d., Tosoh Bioscience, Stuttgart, Germany) was used as a stationary phase. The PDA data were acquired in the 190–320 nm range and the 200 nm signal was extracted. Gradient elution: mobile phase A (CH_3_CN); mobile phase B (H_2_O); gradient, 0–2 min, 20% B; 2–15 min, 20%–60% B; 15–16 min, 60% B, 16–18 min, 60%–20% B, 18–21 min, 20% B (column equilibration). Flow rate was 1 mL/min at 25 °C. The experiment was monitored for 24 h. Retention times were found as follows: NAG-thiazoline **1**, 6.1 min; α-GlcNAc-SH **11a**, 6.8 min; β-GlcNAc-SH **11b**, 7.1 min; oxidation products, 9–13 min. *In situ* reduction of the products of spontaneous oxidation was performed by adding dithiothreitol (1 M) to the reaction mixture in the final concentration of 100 mM and monitored by HPLC and NMR.

### 3.6. Analysis of Decomposition Products of NAG-Thiazoline **1**

NAG-thiazoline (**1**; 200 mg, 0.91 mmol) was dissolved in 50 mM K_2_HPO_4_/KH_2_PO_4_ buffer pH 6.5 (4 mL) and incubated at 35 °C and 850 rpm for 5 h. Then, the reaction volume was reduced to 2.5 mL *in vacuo* and the reaction mixture was loaded on a Biogel P-2 column (BioRad, Prague, Czech Republic) in water, flow rate 12 mL/h. A mixture of oxidation products was isolated (20 mg). This mixture was further practically inseparable since the compounds coexisted in equilibrium. Analysis by MS revealed the presence of masses corresponding to C_8_H_15_NO_5_S calcd. 237.07, *m/z* [M–H]^−^ found 236.0; and to C_16_H_28_N_2_O_10_S_2_ (tentative structure GlcNAc-S-(1↔1)-S-GlcNAc) calc. 472.1, *m/z* [M–H]^−^ found 471.0 (HPLC and MS are presented in the Supplementary Information). After application of dithiothreitol, the oxidation mixture was quickly reduced to the anomeric mixture of 2-acetamido-2-deoxy-1-thio-d-glucopyranose **11a** (α) and **11b** (β). (**11a**) ^1^H-NMR (700.13 MHz, D_2_O): 1.749 (3H, s, 2-Ac), 3.136 (1H, dd, *J* = 10.1, 8.5 Hz, H-4), 3.507 (2H, d, *J* = 3.7 Hz, H-6), 3.565 (1H, dd, *J* = 10.5, 8.5 Hz, H-3), 3.685 (1H, dd, *J* = 10.5, 5.0 Hz, H-2), 3.862 (1H, dt, *J* = 10.1, 3.7 Hz, H-5), 5.272 (1H, d, *J* = 5.0 Hz, H-1), ^13^C-NMR (176.07 MHz, D_2_O): 22.45 (2-Ac), 55.29 (C-2), 60.93 (C-6), 70.73 (C-4), 71.37 (C-5), 71.56 (C-3), 80.37 (C-1), 174.27 (CO). (**11b**): ^1^H-NMR (700.13 MHz, D_2_O): 1.742 (3H, s, 2-Ac), 3.124 (1H, m, H-5), 3.150 (1H, m, H-4), 3.174 (1H, m, H-3), 3.352 (1H, dd, *J* = 10.0, 9.9 Hz, H-2), 3.396 (1H, dd, *J* = 12.4, 5.5 Hz, H-6u), 3.577 (1H, dd, *J* = 12.4, 2.0 Hz, H-6d), 4.385 (1H, d, *J* = 9.9 Hz, H-1), ^13^C-NMR (176.07 MHz, D_2_O): 22.57 (2-Ac), 59.72 (C-2), 61.25 (C-6), 70.18 (C-4), 75.78 (C-3), 80.23 (C-5), 80.69 (C-1), 174.63 (C=O). Results of NMR analysis of oxidation product mixture are not in contradiction with the structure GlcNAc-S-(1↔1)-S-GlcNAc as proposed from the MS measurement.

### 3.7. Enzymes

β-*N*-Acetylhexosaminidase from *Talaromyces flavus* was extracellularly expressed in *Pichia pastoris* and purified from the culture media by cation exchange chromatography as described previously [12]. The gene of β-*N*-acetylhexosaminidase from *Streptomyces plicatus* (GenBank ID: AF063001.3) was prepared synthetically as a His_6_-tagged fusion protein (Geneart, Regensburg, Germany) and cloned into the bacterial expression vector pET-15b *via* the *Nde*I and *Bam*HI restriction sites. The gene was intracellularly expressed in *E. coli* BL21(DE3) pLysS strain (Promega, Prague, Czech Republic) under the IPTG (0.5 mM) induction. After 16 h of cultivation at 25 °C, the cells were harvested by centrifugation (4,000 *×g*, 15 min) and resuspended in a lysis buffer (50 mM Tris/HCl, pH 7) with 50 mM NaCl, 4 mM MgCl_2_, 0.5% Triton X-100, 1 mM phenylmethylsulfonyl fluoride and lysozyme (1 mg/mL). The cells were then incubated for 30 min at 37 °C and disrupted with ultrasound (6 × 1 min). After centrifugation (13,500 *×g*, 10 min) the supernatant was loaded onto Ni-NTA agarose column (Qiagen by Dynex, Bustehrad, Czech Republic). The column was equilibrated with 50 mM Tris/HCl buffer with 150 mM KCl and 10 mM imidazole, pH 7.4. The fusion protein was eluted from the column using 50 mM Tris/HCl buffer supplemented with 150 mM KCl and 250 mM imidazole, pH 7.4. The purity of the fractions was determined by 10% SDS-PAGE.

The genes of *O*-GlcNAcase from *Bacteroides thetaiotaomicron* and human *O*-GlcNAcase were obtained from Prof. D. Vocadlo (SFU, Burnaby, BC, Canada)*.* The genes were intracellularly expressed in *E. coli* BL21(DE3) pLysS strain under the induction by IPTG (0.5 mM). After 4 h of cultivation at 16 °C, the cells were harvested by centrifugation (4,000 *×g*, 15 min). The procedure for the isolation of both *O*-GlcNAcases was analogous to the purification procedure for β-*N*-acetylhexosaminidase from *Streptomyces plicatus* as described above*.*

### 3.8. β-*N*-Acetylhexosaminidase and *O*-GlcNAcase Activity and Inhibition Assays

β-*N*-Acetylhexosaminidase and *O*-GlcNAcase activities were assayed in end-point experiments using *p*-nitrophenyl 2-acetamido-2-deoxy-β-d-glucopyranoside (*p*NP-β-GlcNAc; Sigma Aldrich) as a substrate; starting concentration 2 mM. One unit of enzymatic activity was defined as the amount of enzyme releasing 1 μmol of *p*-nitrophenol per minute in 50 mM citrate-phosphate buffer at pH 7.0 and 35 °C. After incubation of the reaction mixture at 35 °C for 10 min, the liberated *p-*nitrophenol was determined spectrophotometrically at 420 nm under alkaline conditions (50 μL of the reaction mixture was added to 1 mL of 0.1 M Na_2_CO_3_).

Michaelis-Menten kinetics of β-*N*-acetylglucosaminidase reactions was measured in a kinetic assay at 348 nm (isosbestic point of *p*-nitrophenol), using *p*NP-β-GlcNAc as substrate with its concentration in the range of 0.04–0.5 mM (50 mM citrate-phosphate buffer pH 7.0, 25 °C); in the inhibition assays the concentration of the inhibitors ranged from 0.001 to 0.5 mM for *O*-GlcNAcases and from 0.05 to 2 mM for β-*N*-acetylhexosaminidases. All data were measured in five parallels; the non-linear regression was calculated using Enzfitter (Biosoft, UK). Lineweaver-Burk plots for the individual experiments are shown in the Supplementary information.

### 3.9. Molecular Modeling and Docking

C-6-azido-NAG-thiazoline (**2**) was constructed in YASARA by modification of the crystal structure of NAG-thiazoline (**1**) extracted from the enzyme/inhibitor complex of β-*N*-acetylhexosaminidase from *Streptomyces plicatus* with NAG-thiazoline (pdb code: 1hp5) [13]. Quantum calculations to determine the partial charges were performed in Gaussian 03 [14] at the B3LYP/6-31G(p,d) level of theory. The resulting charges were converted to RESP charges by Antechamber [15] and included in the molecular topology to be used in YASARA. *p*NP-β-GlcNAc was constructed in YASARA by modification of di-*N*-acetyl-d-glucosamine (chitobiose), extracted from the crystal structure of chitobiase from *Serratia marcescens* (pdb code: 1qbb) [16].

The substrate *p*NP-β-GlcNAc, inhibitor C-6-azido-NAG-thiazoline (**2**) and NAG-thiazoline (**1**) were docked in the active site of selected β-*N*-acetylhexosaminidases (GH20) and β-*N*-acetylglucosaminidases (GH84) using rigid docking in AutoDock 4.2.3 [17] to accommodate them properly in the active site of enzymes, using a grid space of 0.275 Å, the Lamarckian genetic algorithm [18], and 100 docking runs). Structures of enzymes used for docking are listed as follows: crystal structure of *O*-GlcNAcase from *Bacteroides thetaiotaomicron* (pdb code: 2chn) [19]; model of human *O*-GlcNAcase [20]; crystal structure of β-*N*-acetylhexosaminidase from *Streptomyces plicatus* (pdb code: 1hp5) [13] and the model of β-*N*-acetylhexosaminidase from *Talaromyces flavus* (will be published elsewhere). The obtained complexes with best docking scores were used for molecular dynamics simulation in water with YASARA (isothermal–isobaric ensemble (NPT), counter ions to reach neutrality, YASARA 2 force field [21], periodic boundary condition, long range electrostatics treated by Particle Mesh Ewald method [22]). Molecular dynamics simulations were run for more than 10 ns and analyzed. Root mean square deviation of all C-alpha and of active site amino acid residues was used to asses if complexes are equilibrated. Additionally, the interaction energy from the force field was calculated during the whole simulation with YASARA to monitor if the energy reaches a stable plateau and thus indicates that the substrate/inhibitor interaction with the enzyme is stable. Finally, a set of representative structures of stable substrate/inhibitor-enzyme complexes from the last 2 ns of the equilibrated production period of the simulations was used for binding energy calculations by AutoDock and for analysis with YASARA. Interactions in the active site were shown with LigPlot+ [23] and YASARA.

## 4. Conclusions

A series of novel derivatives of GlcNAc-thiazolines substituted (compounds **2**–**8**) or dimerized at C-6 (compounds **9**–**10**) bearing azido or triazole moieties were designed as potential β-*N*-acetylhexosaminidase inhibitors and synthesized. New compounds were tested for their inhibitory activities with GH20 β-*N*-acetylhexosaminidases and GH84 β-*N*-acetylglucosaminidases. One bacterial and one eukaryotic model enzyme of each glycosidase family were selected for the inhibition study: GH20 β-*N*-acetylhexosaminidases from *Streptomyces plicatus* (bacterial) and *Talaromyces flavus* (fungal) and GH84 β-*N*-acetylglucosaminidases from *Bacteroides thetaiotaomicron* (bacterial) and humans. While all the triazole-substituted NAG-thiazolines **3**–**10** lacked inhibition activity with the enzymes used, the C-6-azido-NAG-thiazoline (**2**) inhibited most of the enzymes in a moderate way. Elaborated kinetic study was performed to compare both the original **1** and the new inhibitor **2**. Molecular docking of C-6-azido-NAG-thiazoline **2** into the active site of the respective enzymes and consequent molecular dynamics simulations of the resulting complexes were performed to corroborate the conclusions obtained from the “wet” experiments with a molecular explanation. Generally, the affinity of β-*N*-acetylhexosaminidases to modified NAG-thiazoline **2** is lower than to the substrate having more hydrogen bonding partners. A detailed stability study with unsubstituted GlcNAc-thiazoline **1** has shown its decomposition at pH < 6 resulting in the thiazoline ring opening yielding 2-acetamido-2-deoxy-1-thio-α/β-d-glucopyranoses **11a**,**b**, which presumably dimerize oxidatively into S-S linked dimers. Tentative structures of the products were supported by mass spectrometry and NMR measurements. Decomposition products proved to have no inhibitory activity towards β-*N*-acetylhexosaminidase (*T. flavus*).

## Supplementary Materials

Supplementary materials can be accessed at: http://www.mdpi.com/1420-3049/19/3/3471/s1.
