# Supplementary material for: Inhibition of GlcNAc-Processing Glycosidases by C-6-Azido-NAG-Thiazoline and Its Derivatives
Source: Molecules. 2014 Mar 20;19(3):3471–88. doi: 10.3390/molecules19033471 (PMC6271965; doi:10.3390/molecules19033471)

Supplementary File

1. ^{1}H and ^{13}C-NMR Spectra of the New Compounds **2–10**—General Methods

NMR spectra were recorded on a Bruker Avance III 400 MHz spectrometer (400.00 MHz for ^{1}H, 100.58 MHz for ^{13}C at 30 °C in CD_3OD – compounds **2**–**10**) and a Bruker Avance III 700 MHz spectrometer (700.13 MHz for ^{1}H, 176.07 MHz for ^{13}C at 30 °C). Residual signals of solvent were used as internal standards (δ_H 3.330 ppm, δ_C 49.30 ppm for CD_3OD; δ_H 4.508 ppm for D_2O). Carbon chemical shifts in D_2O were referenced to acetone (δ_C 30.50 ppm). NMR experiments ^{1}H-NMR, ^{13}C-NMR, gCOSY, gHSQC, and gHMBC were performed using the manufacturer's software. ^{1}H-NMR and ^{13}C-NMR spectra were zero filled to fourfold data points and multiplied by window function before Fourier transformation. Two-parameter double-exponential Lorentz-Gauss function was applied for ^{1}H to improve resolution and line broadening (1 Hz) was applied to get better ^{13}C signal-to-noise ratio. Chemical shifts are given in δ-scale with digital resolution justifying the reported values to three (δ_H) or two (δ_C) decimal places.

Proton spin systems of thiazoline and triazole-linker moieties were assigned by COSY and by HSQC transferred to carbons; HMBC experiment enabled to join above mentioned partial structures together. Thiazoline structure was proved by the presence of methyl doublet (J = 2.0 or 2.1 Hz) correlated in HMBC to carbons C-1 and C-2. Dimer formation (compounds **9** and **10**) was unambiguously confirmed by the auto-correlation cross peak of the central carbon of the linker (C-3' for **10** and C-5' for **9**).

2. Mass Spectrometry

The exact masses were measured using LTQ Orbitrap XL hybrid mass spectrometer (Thermo Fisher Scientific, Waltham, MA, USA) equipped with an electrospray ion source. The mobile phase consisted of methanol/water (4:1), flow rate 30 µL/min, and the samples were injected using a 2-µL loop. The mass spectra of positively charged ions were internally calibrated using protonated phthalic anhydride as lock mass. Data were acquired and processed using Xcalibur software (Thermo Fisher Scientific).

6-Azido-1,2-dideoxy-2'-methyl-α-D-glucopyrano-[2,1-d]-Δ2'-thiazoline (**2**). HRMS: $C_8H_{12}O_3N_4NaS$ calcd. 267.05223; *m/z* $[M+Na]^+$ found 267.05227 (Figure S1).

Figure S1. Structure, NMR and HRMS spectra of compound **2**.

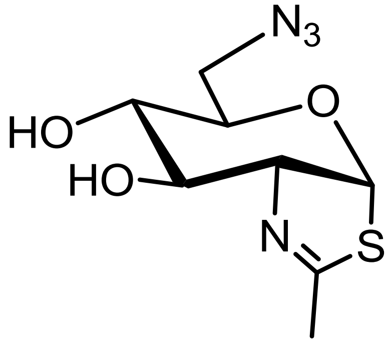

Figure S1. *Cont.*

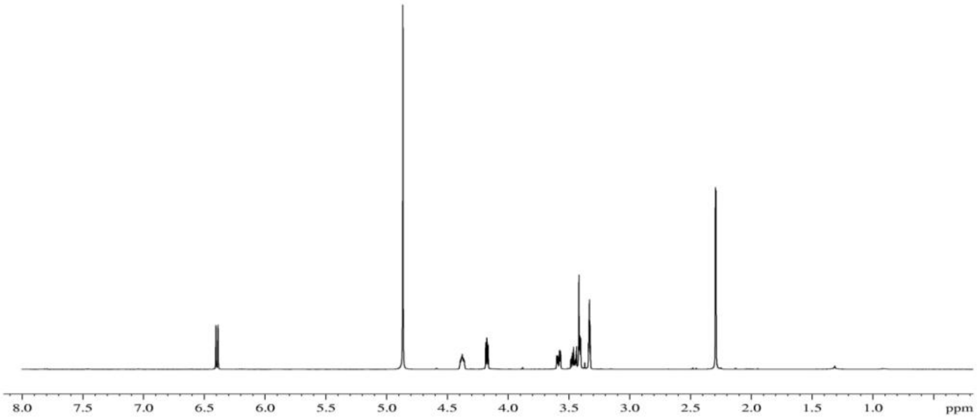

^{1}H-NMR

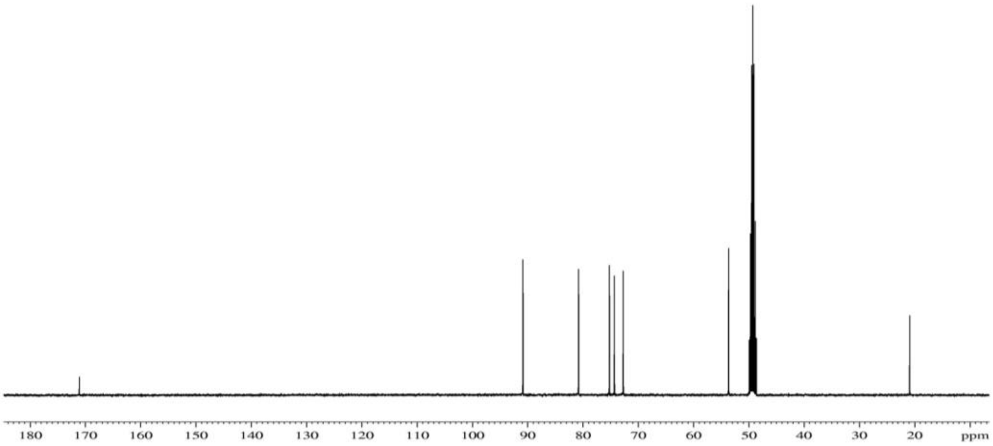

^{13}C-NMR

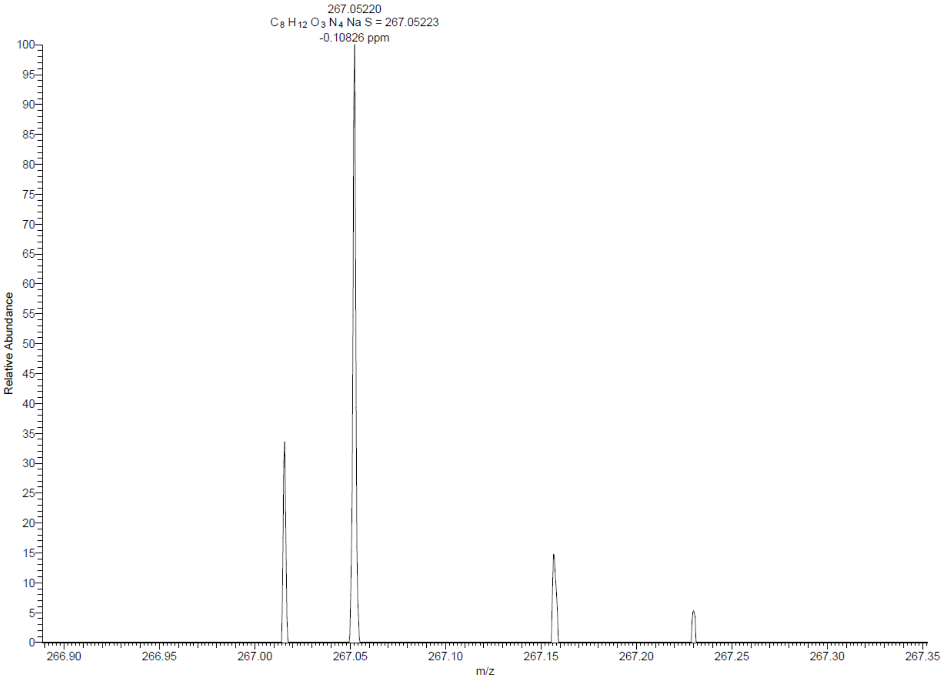

HRMS

6-(4-Butyltriazolyl)-1,2-dideoxy-2'-methyl-α-D-glucopyrano-[2,1-d]-Δ2'-thiazoline (**3**). HRMS: $C_{14}H_{23}O_3N_4S$ calcd. 327.14854; *m/z* $[M+H]^+$ found 327.14848 (Figure S2).

Figure S2. Structure, NMR and HRMS spectra of compound **3**.

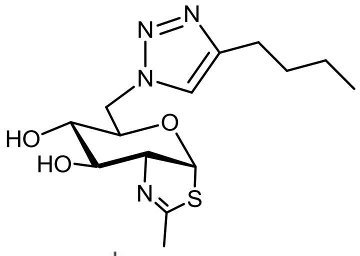

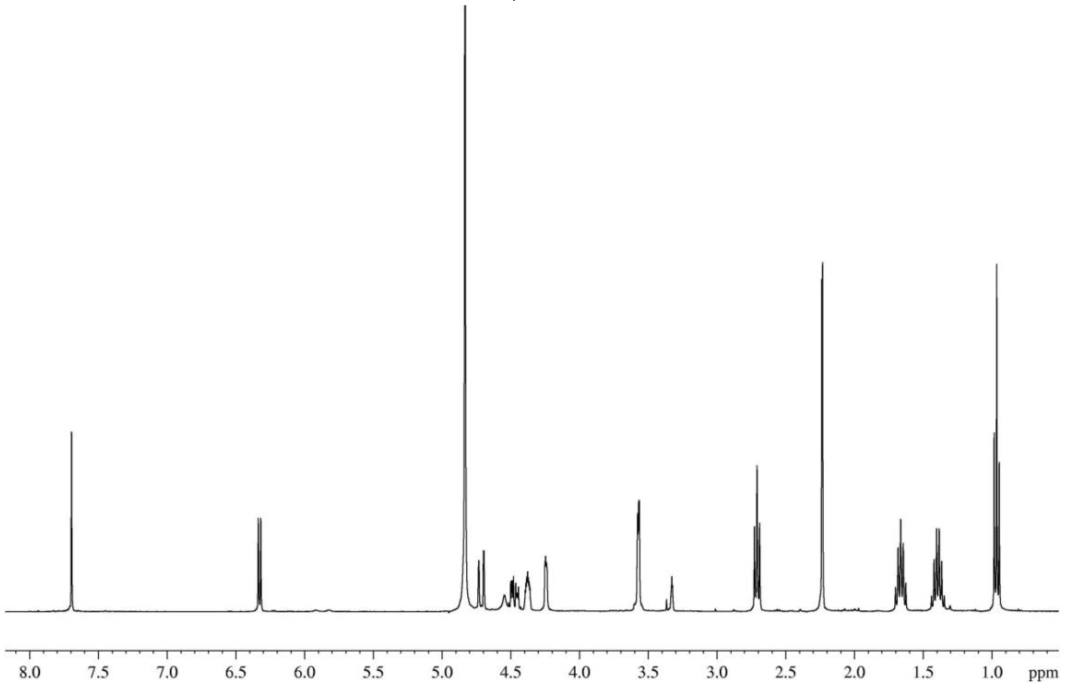

^{1}H-NMR

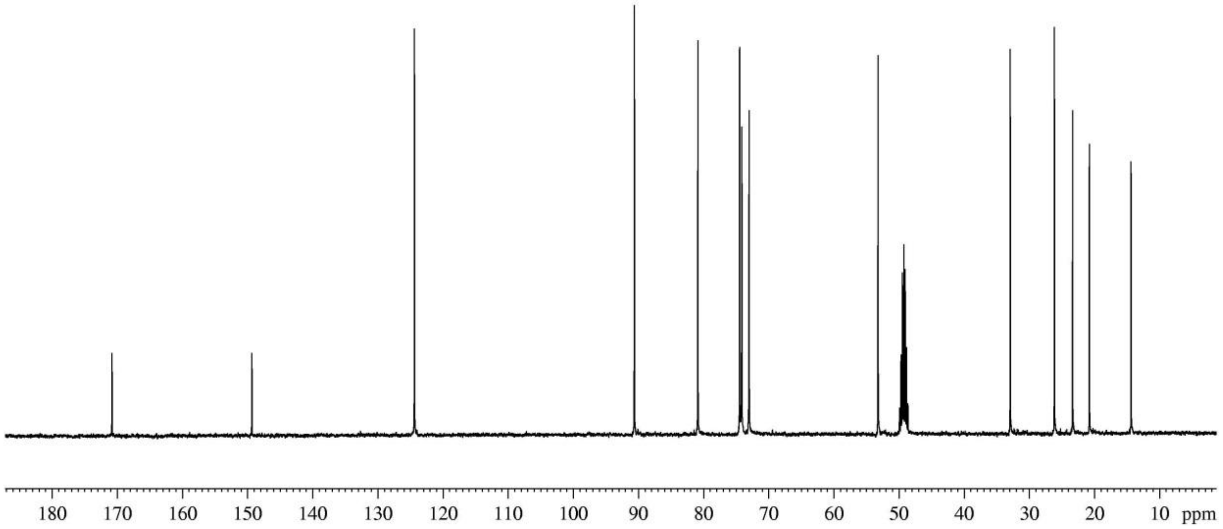

^{13}C-NMR

Figure S2. *Cont.*

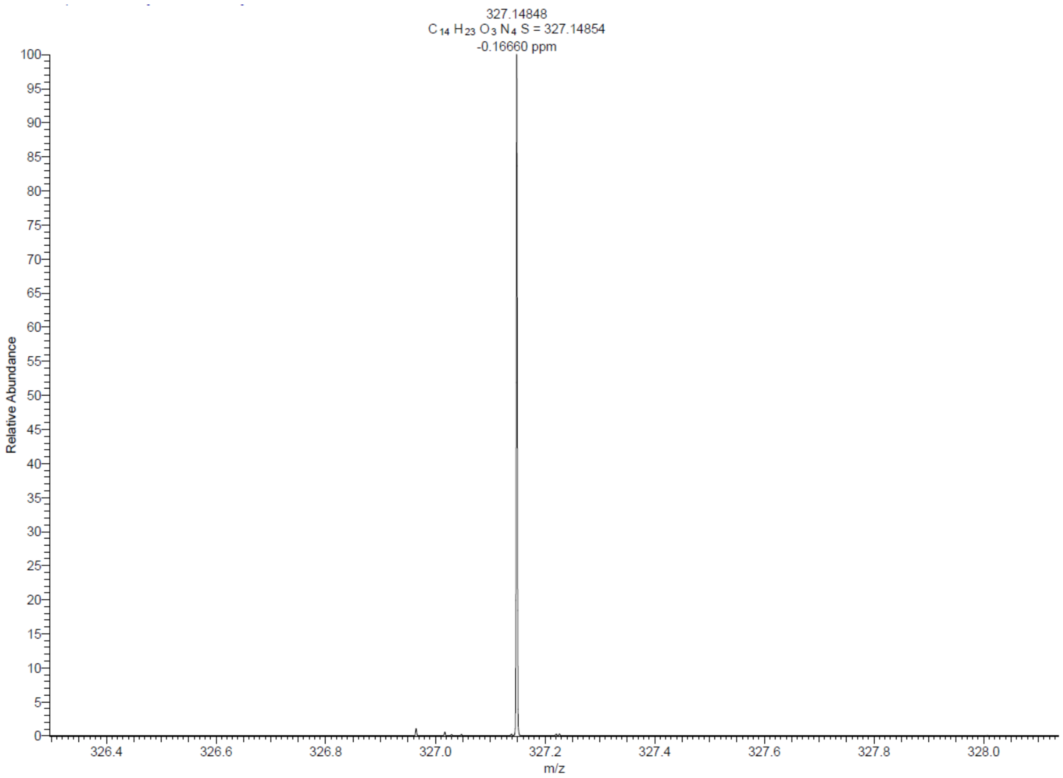

HRMS

1,2-Dideoxy-2'-methyl-6-(4-phenyltriazolyl)-α-D-glucopyrano-[2,1-d]-Δ2'-thiazoline (**4**). HRMS: $C_{16}H_{19}O_3N_4S$ calcd. 347.11724; *m/z* $[M+H]^+$ found 347.11704 (Figure S3).

Figure S3. Structure, NMR and HRMS spectra of compound **4**.

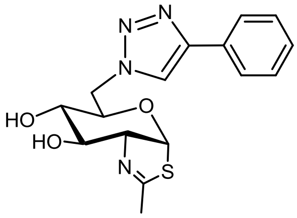

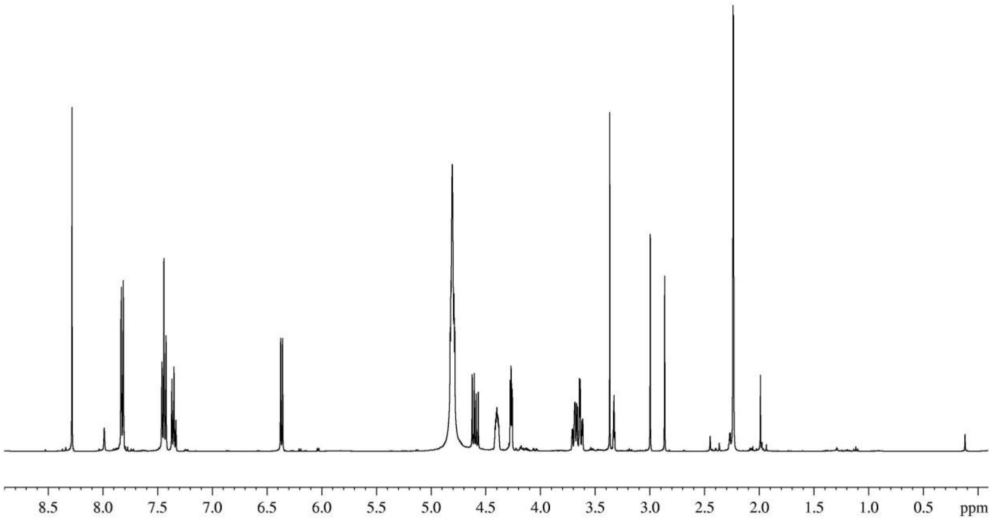

^{1}H-NMR

Figure S3. *Cont.*

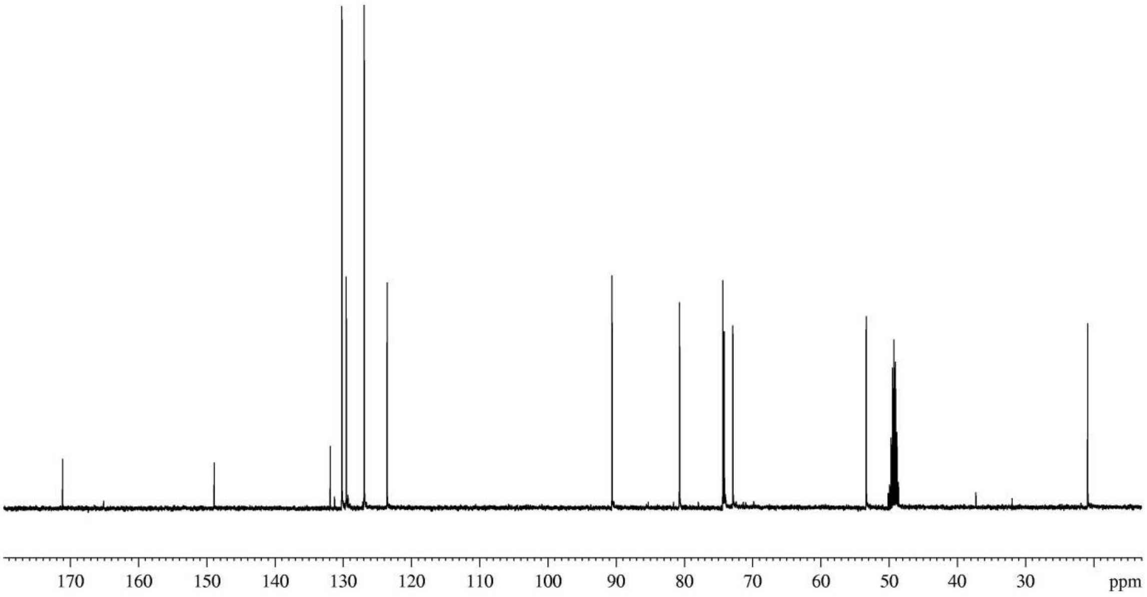

^{13}C-NMR

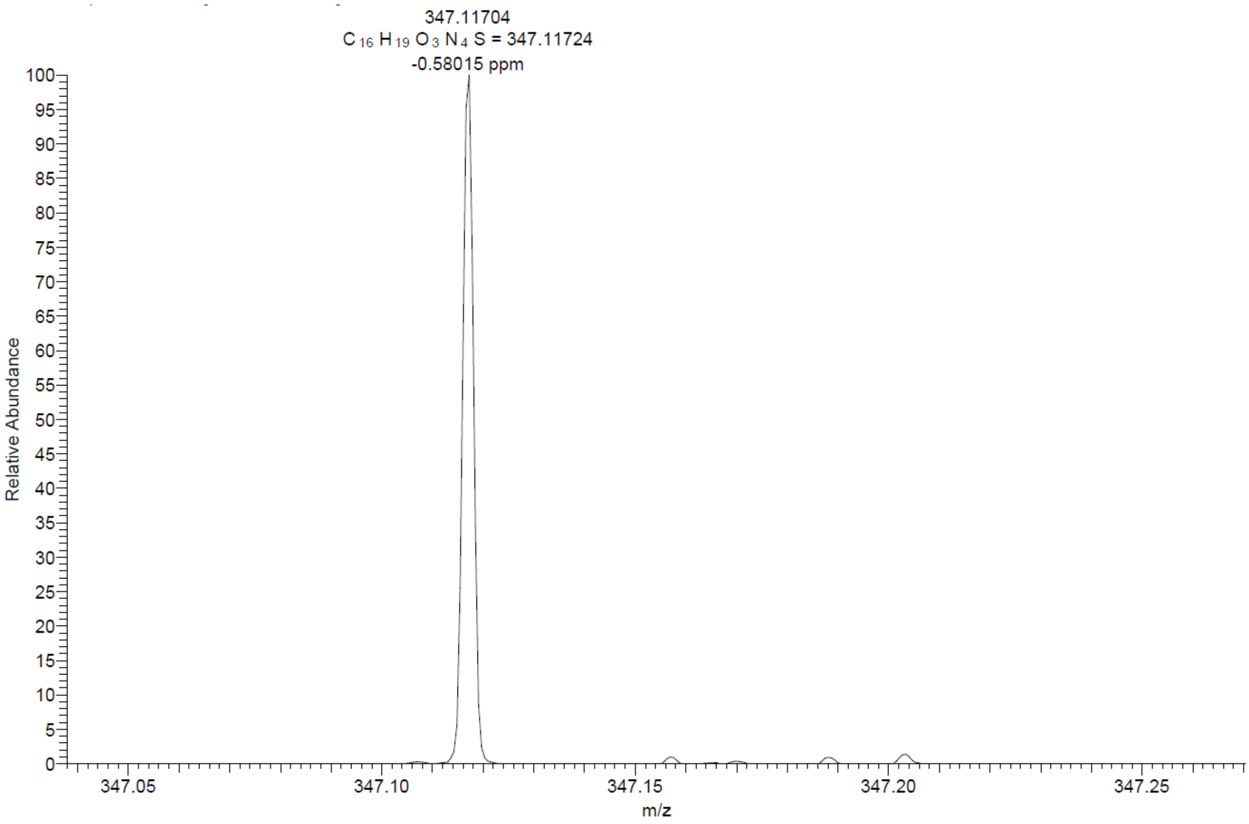

HRMS

1,2-Dideoxy-6-(4-hydroxymethyltriazolyl)-2'-methyl-α-D-glucopyrano-[2,1-d]-Δ2'-thiazoline (**5**). HRMS: $C_{11}H_{17}O_4N_4S$ calcd. 301.09650; *m/z* $[M+H]^+$ found 301.09650 (Figure S4).

Figure S4. Structure, NMR and HRMS spectra of compound **5**.

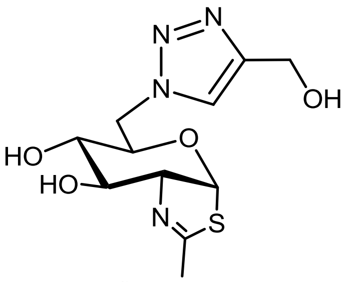

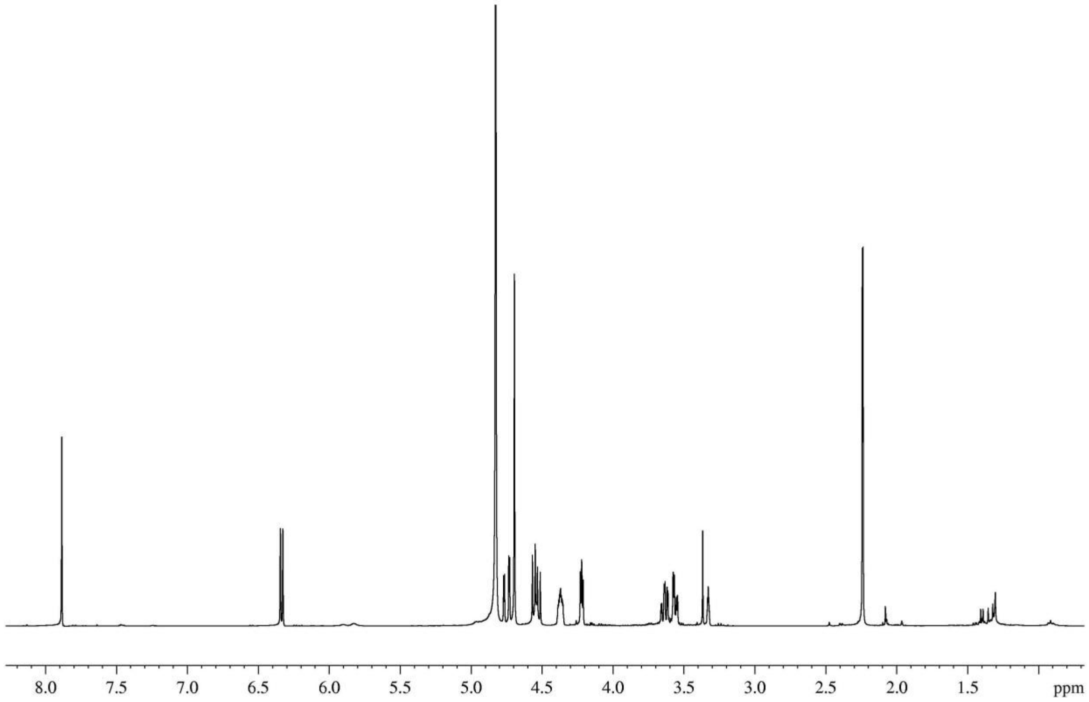

^{1}H-NMR

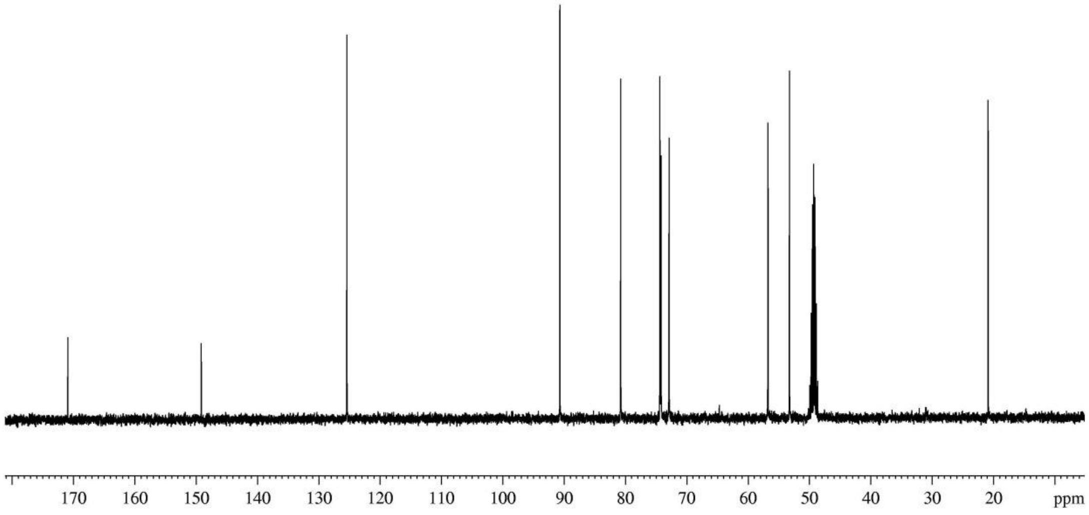

^{13}C-NMR

Figure S4. *Cont.*

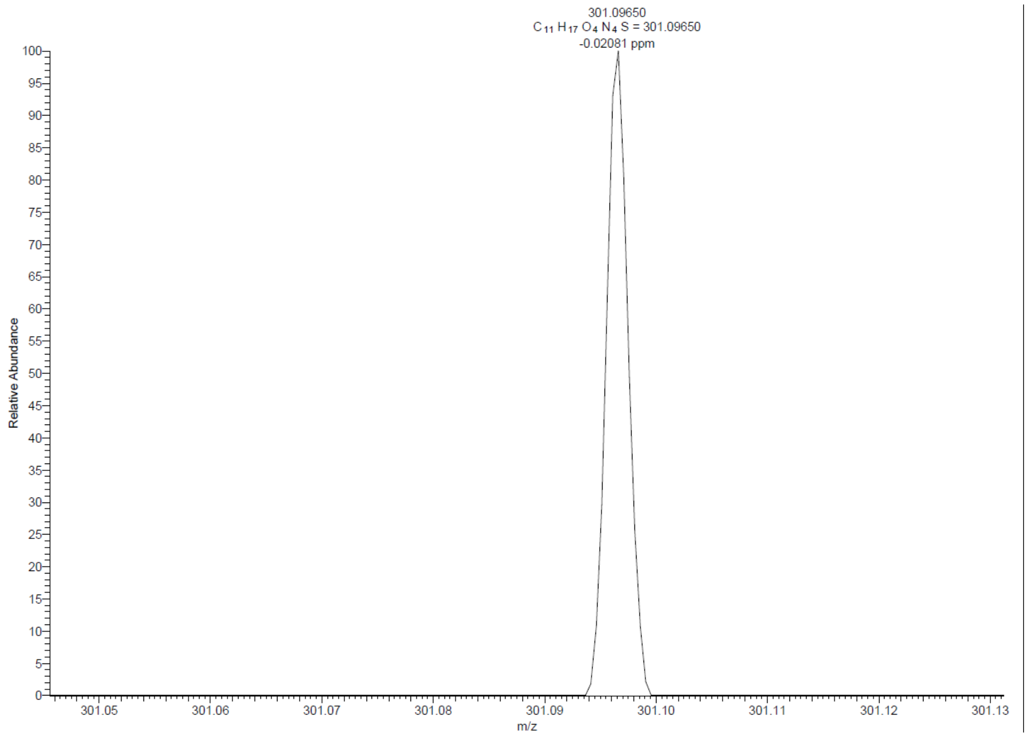

HRMS

1,2-Dideoxy-2'-methyl-6-(4-trimethylsilyltriazolyl)-α-D-glucopyrano-[2,1-d]-Δ2'-thiazoline (**6**). HRMS: $C_{13}H_{23}O_3N_4SSi$ calcd. 343.12546; *m/z* $[M+H]^+$ found 343.12537 (Figure S5).

Figure S5. Structure, NMR and HRMS spectra of compound **6**.

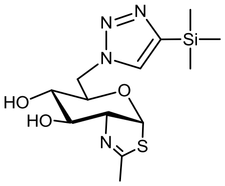

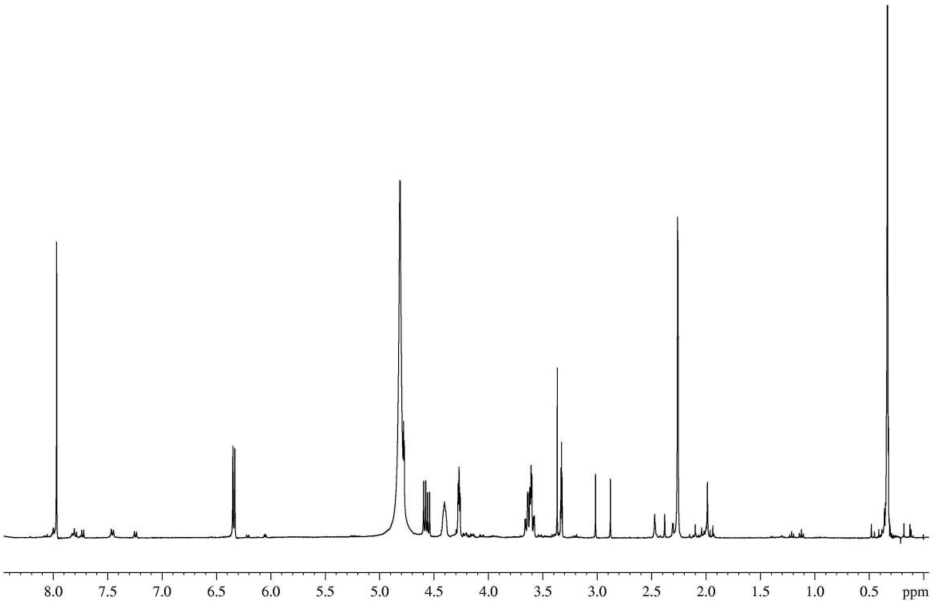

^{1}H-NMR

Figure S5. *Cont.*

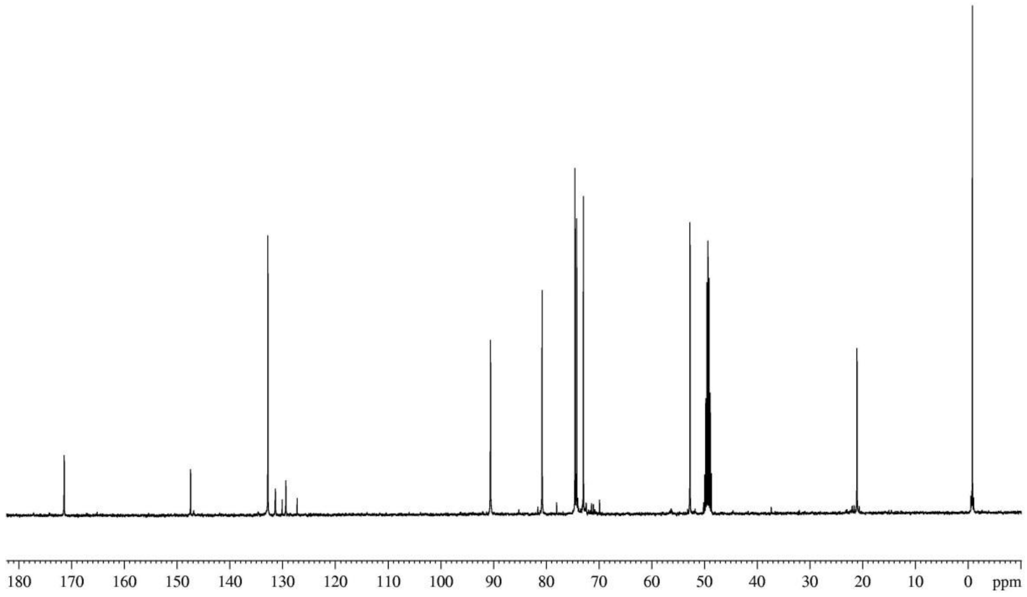

^{13}C-NMR

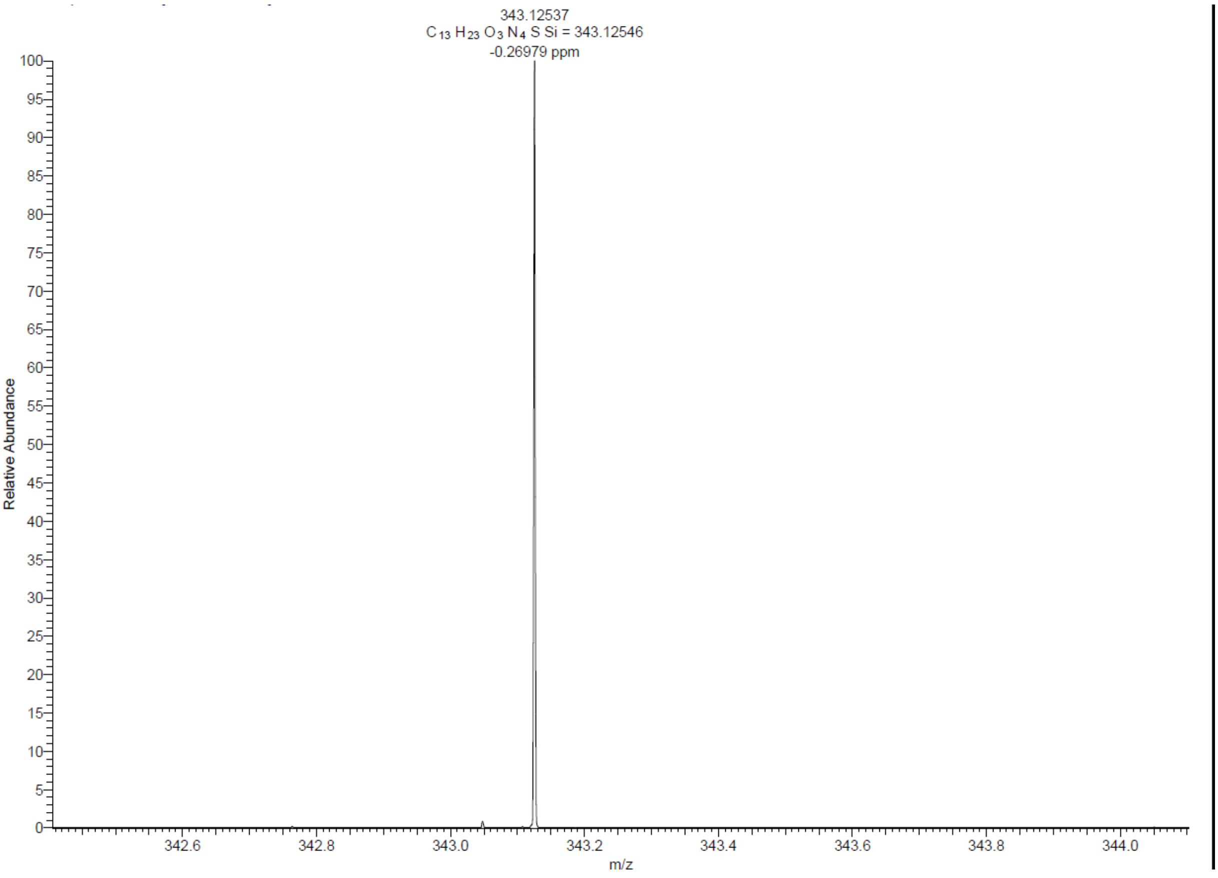

HRMS

1,2-Dideoxy-6-[4-(hex-5-ynyl)triazolyl]-2'-methyl-α-D-glucopyrano-[2,1-d]-Δ2'-thiazoline (**7**). HRMS: $C_{16}H_{22}O_3N_4NaS$ calcd. 373.13048; *m/z* $[M+Na]^+$ found 373.13033 (Figure S6).

Figure S6. Structure, NMR and HRMS spectra of compound **7**.

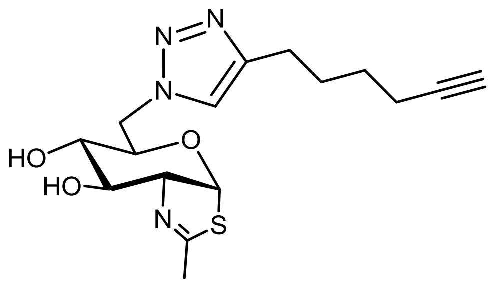

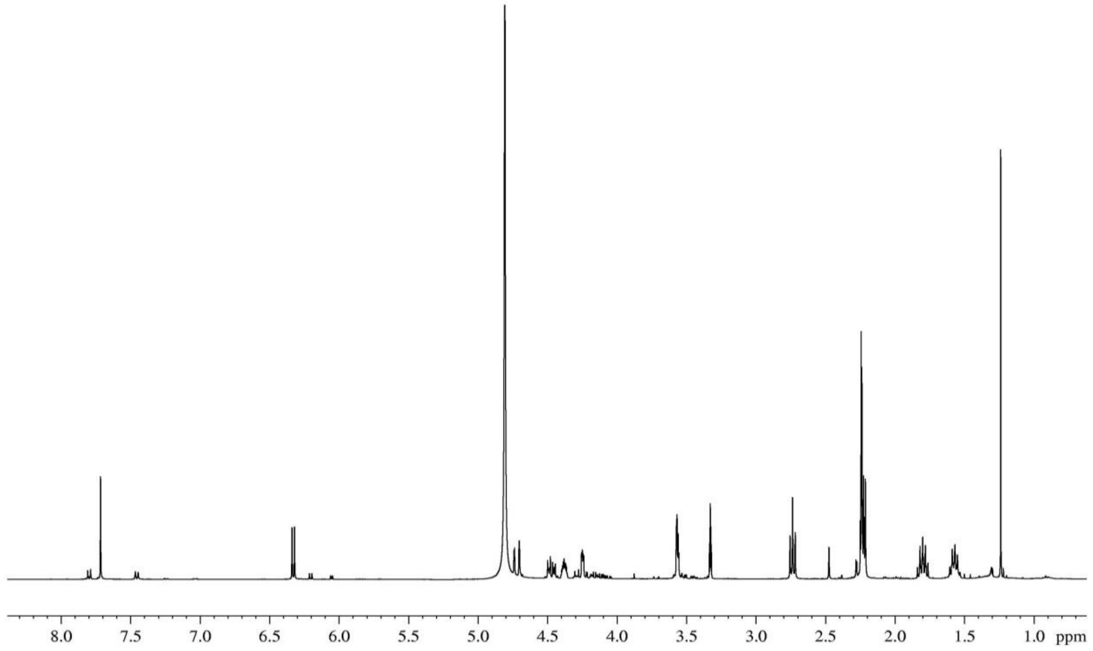

^{1}H-NMR

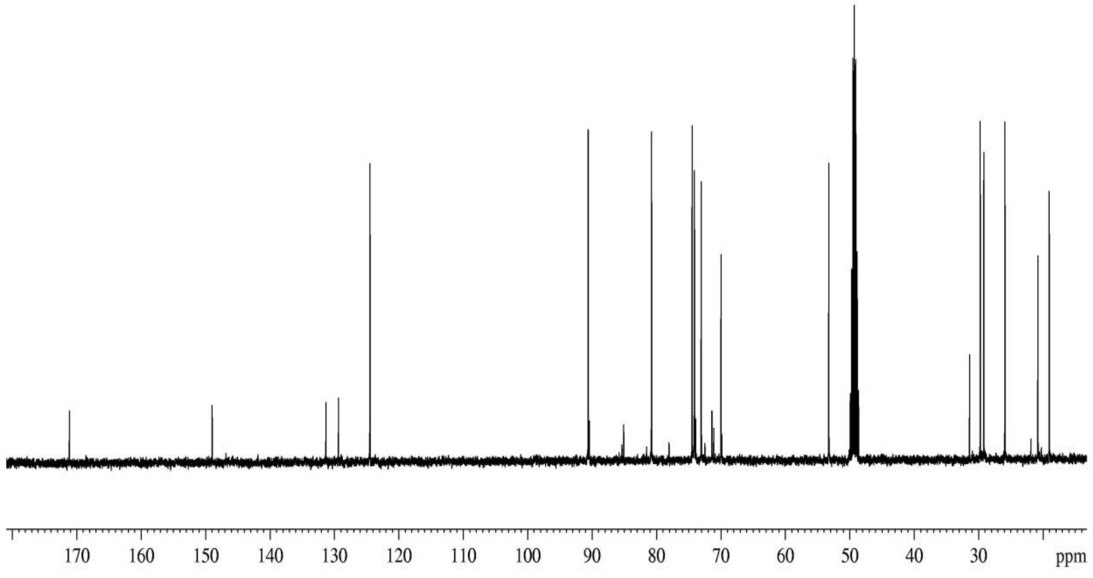

^{13}C-NMR

Figure S6. *Cont.*

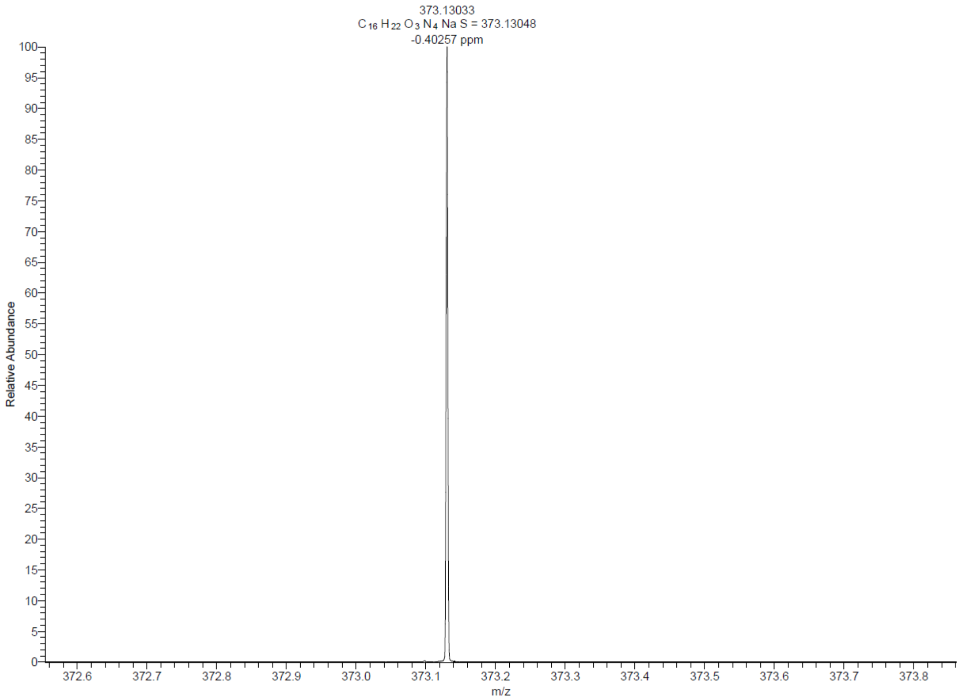

HRMS

1,2-Dideoxy-2'-methyl-6-[4-(propargyloxymethyl)-triazolyl]-α-D-glucopyrano-[2,1-d]-Δ2'-thiazoline (**8**). HRMS: $C_{14}H_{19}O_4N_4S$ calcd. 339.11215; *m/z* $[M+H]^+$ found 339.11203 (Figure S7).

Figure S7. Structure, NMR and HRMS spectra of compound **8**.

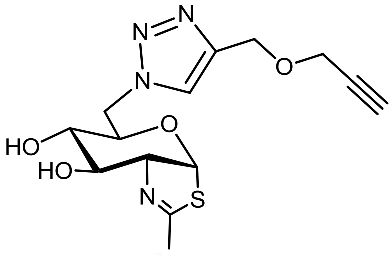

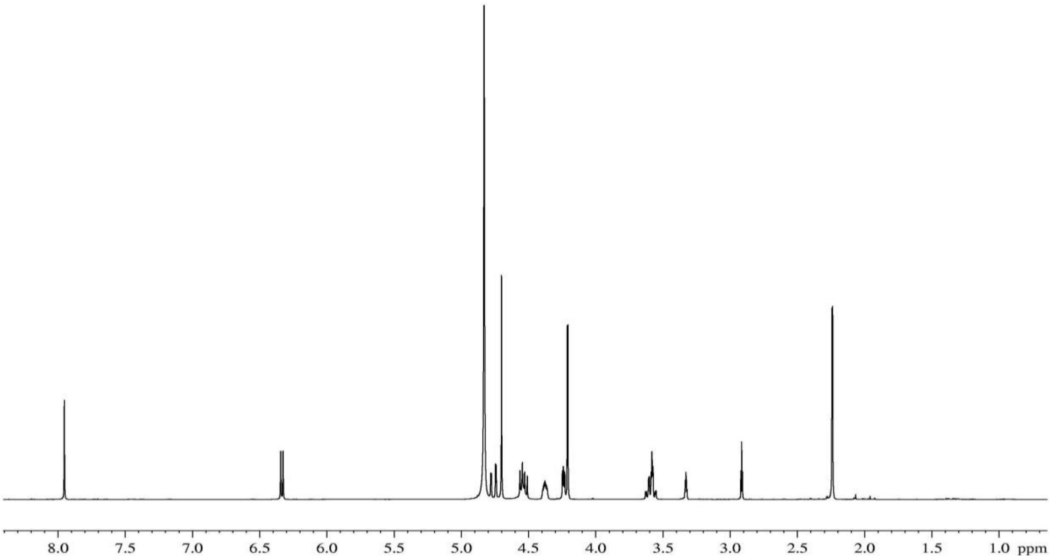

^{1}H-NMR

Figure S7. *Cont.*

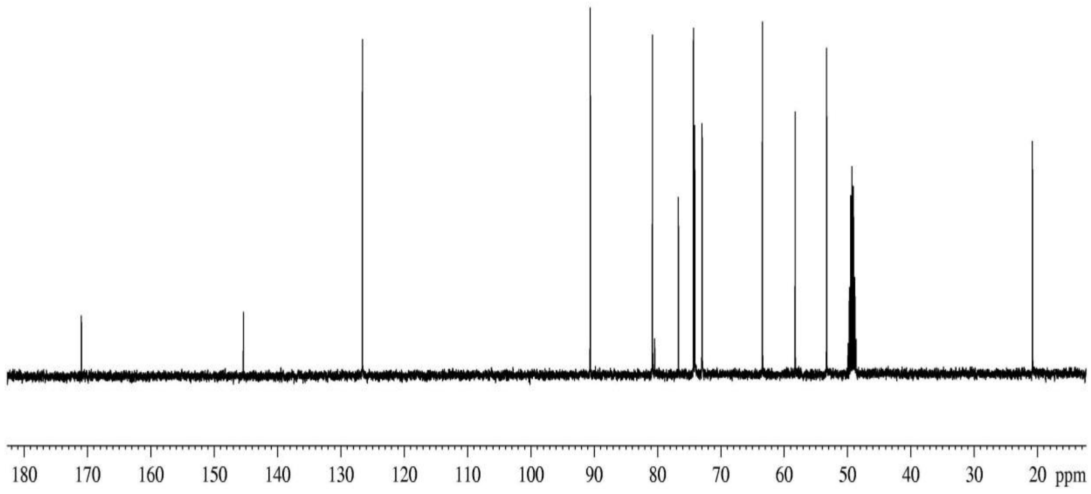

^{13}C-NMR

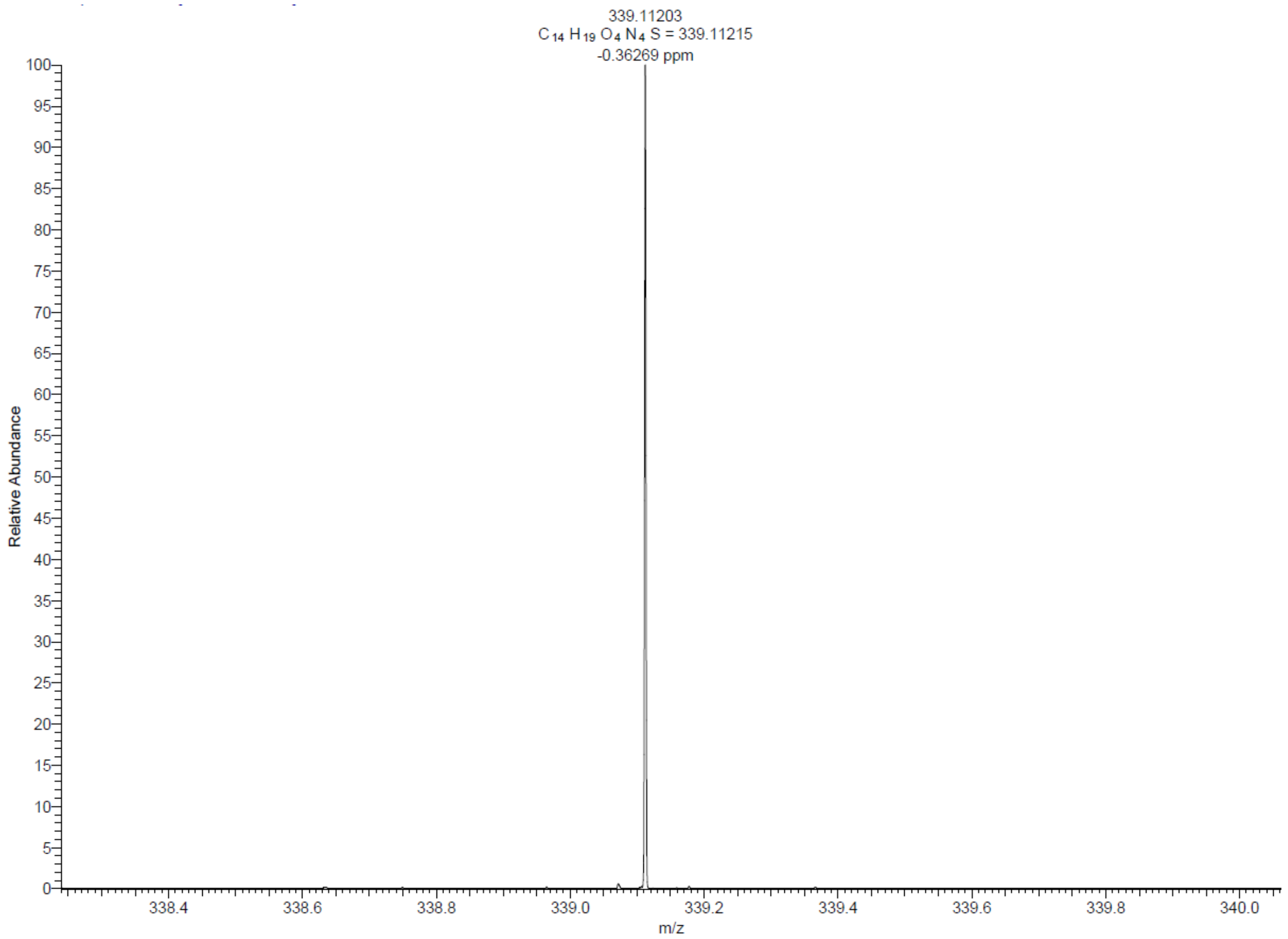

HRMS

1,4-Bis[(1,2-dideoxy-2'-methyl-α-D-glucopyrano-[2,1-d]-Δ2'-thiazolin-6-yl)-triazol-4-yl]-butane (**9**). HRMS: $C_{24}H_{35}O_6N_8S_2$ calcd. 595.21155; *m/z* $[M+H]^+$ found 595.21133 (Figure S8).

Figure S8. Structure, NMR and HRMS spectra of compound **9**.

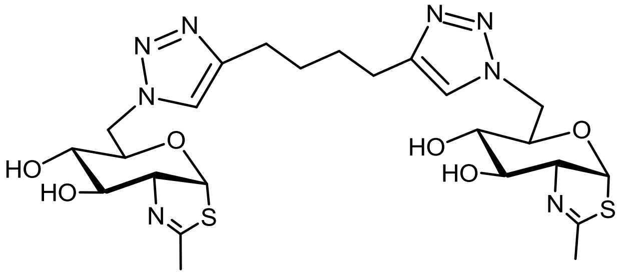

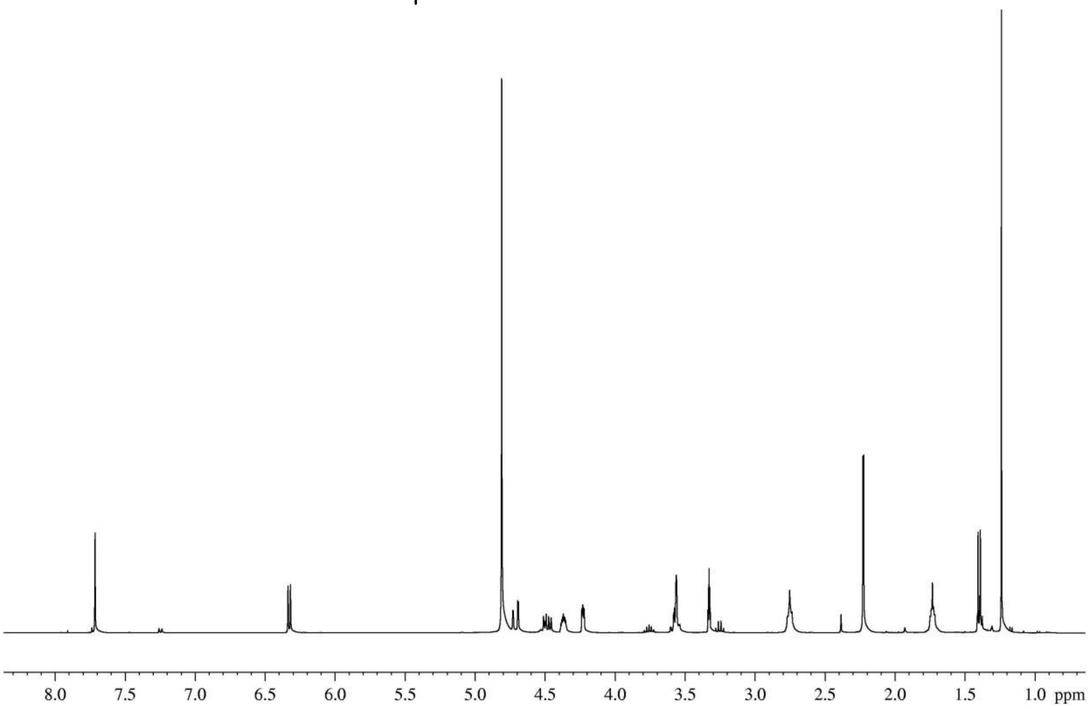

^{1}H-NMR

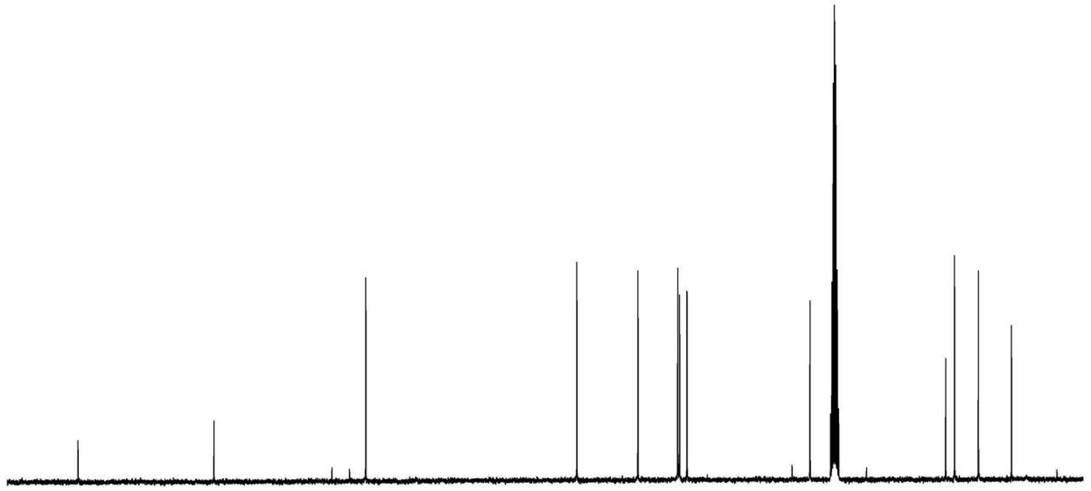

^{13}C-NMR

Figure S8. *Cont.*

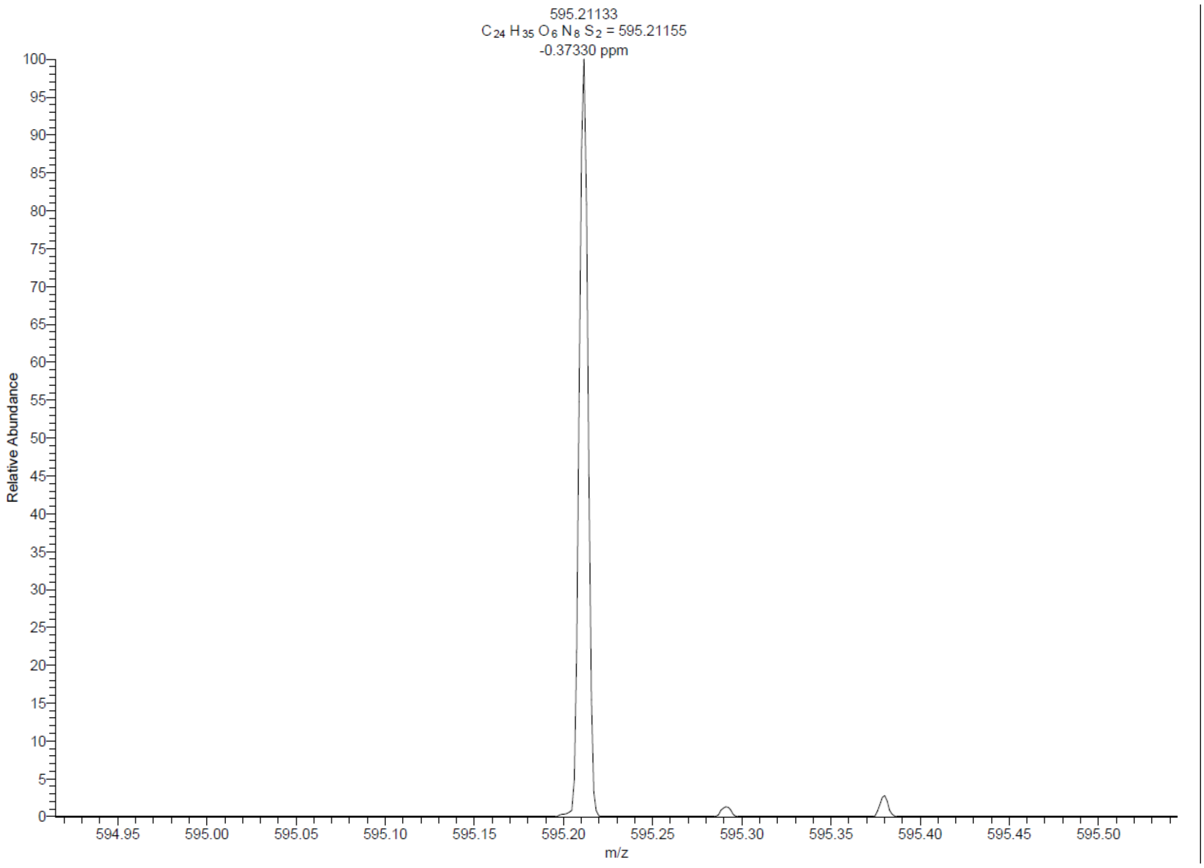

HRMS

Bis{[(1,2-dideoxy-2'-methyl-α-D-glucopyrano-[2,1-d]-Δ2'-thiazoline-6-yl)-triazol-4-yl]-methyl} ether (**10**). HRMS: $C_{22}H_{31}O_7N_8S_2$ calcd. 583.17516; *m/z* $[M+H]^+$ found 583.17503 (Figure S9).

Figure S9. Structure, NMR and HRMS spectra of compound **10**.

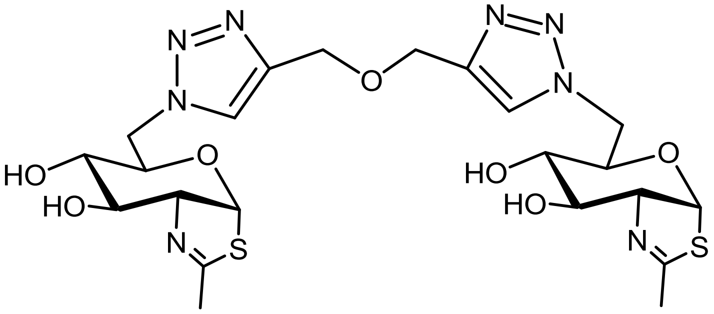

Figure S9. *Cont.*

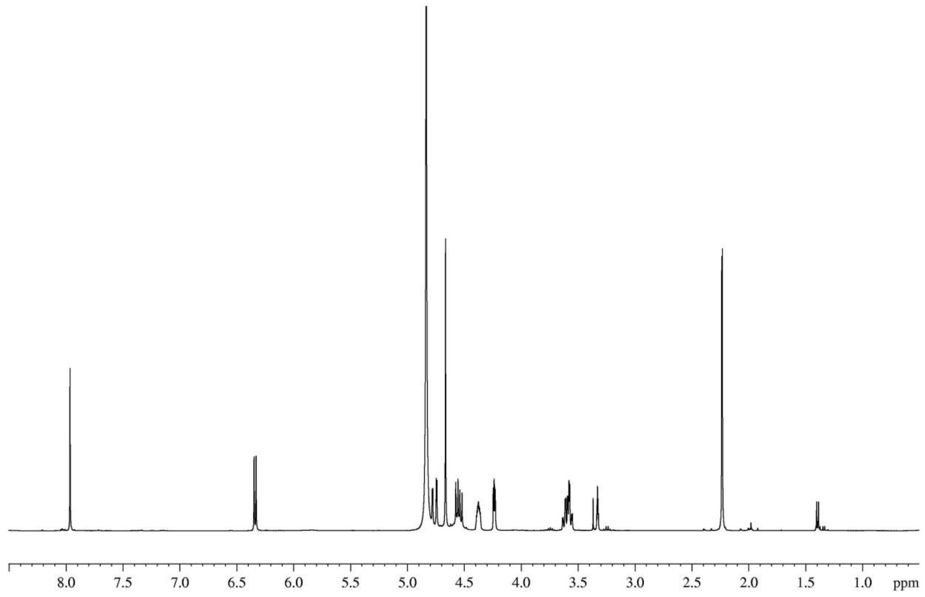

^{1}H-NMR

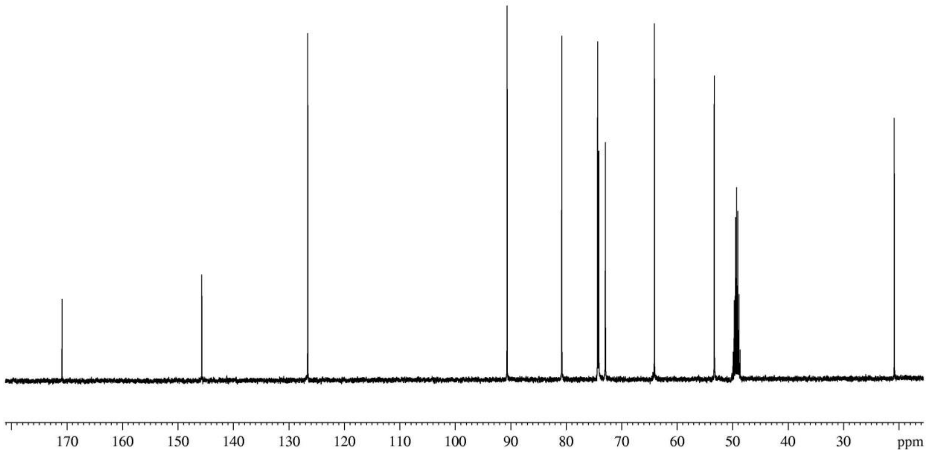

^{13}C-NMR

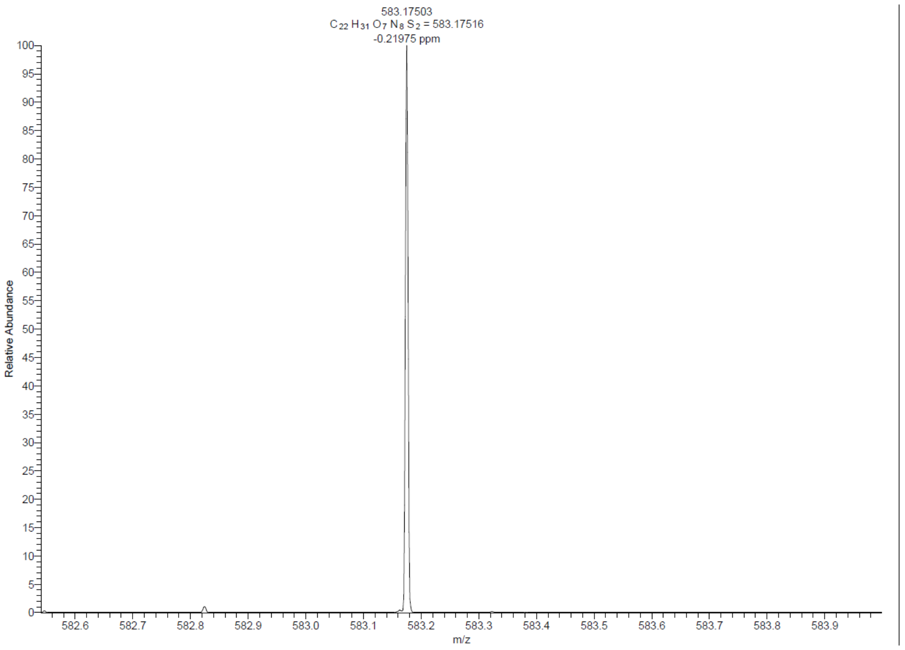

HRMS

3. Mass Spectrum of the Mixture after NAG-Thiazoline Decomposition

Analysis by MS revealed the presence of masses corresponding to $C_8H_{15}NO_5S$ (**11a** and **b**) calcd. 237.07, *m/z* $[M\text{-}H]^-$ found 236.0; and to $C_{16}H_{28}N_2O_{10}S_2$ (tentative structure GlcNAc-S-(1↔1)-S-GlcNAc; **12**) calcd. for $C_{16}H_{28}N_2O_{10}S_2$ 472.120, *m/z* $[M\text{-}H]^-$ found 471.0 (Figure S10).

Figure S10. Mass spectrum of the mixture after NAG-thiazoline decomposition.

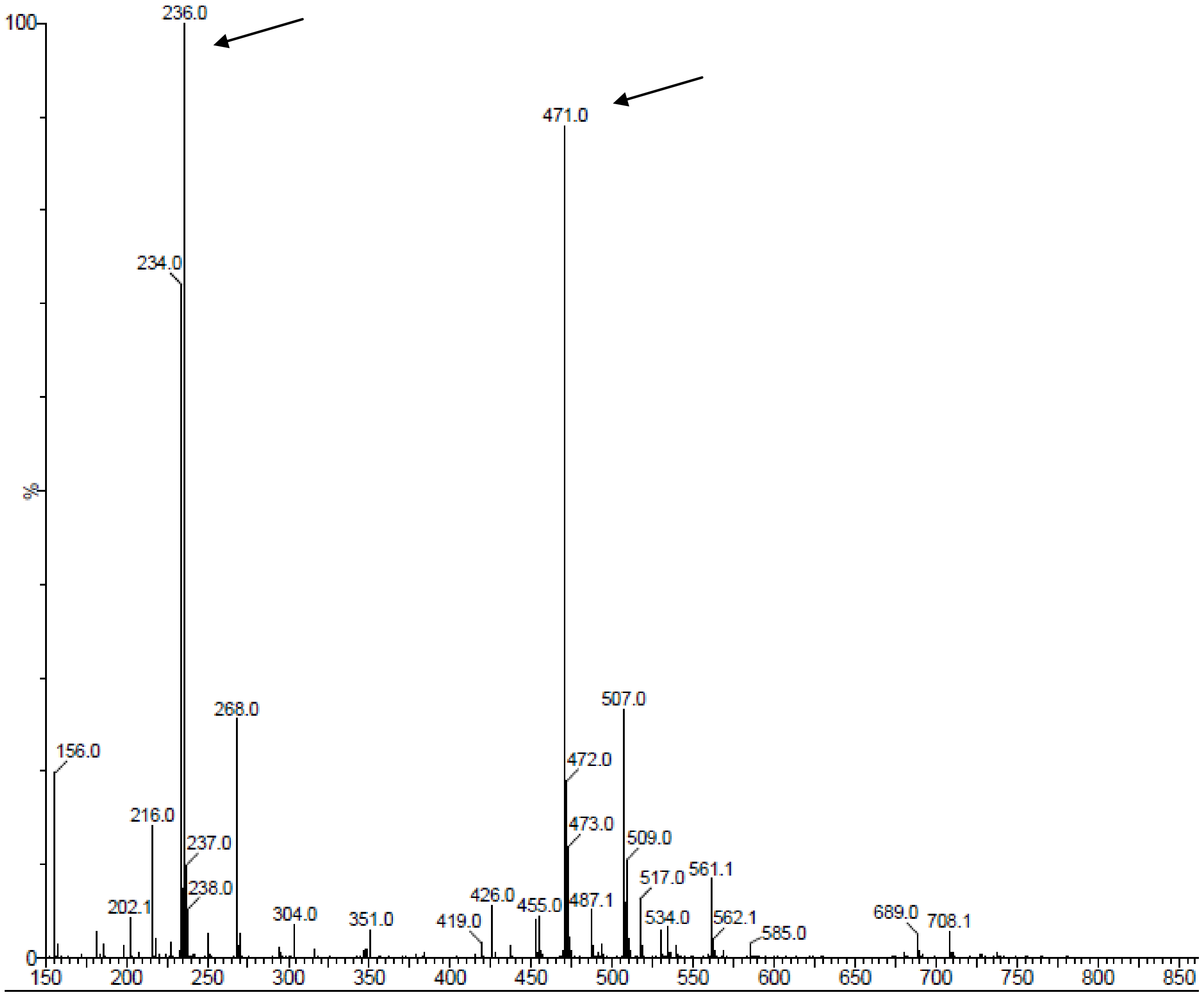

4. HPLC Chromatogram of the Mixture after NAG-Thiazoline Decomposition

Chromatography was carried out on the Shimadzu Prominence UFLC system (Kyoto, JP) consisting of DGU-20A mobile phase degasser, two LC-20AD solvent delivery units, SIL-20ACHT cooling autosampler, CTO-10AS column oven and SPD-M20A diode array detector. The HILIC column TSKgel Amide-80 (250 × 4.6 mm i.d., Tosoh Bioscience, Stuttgart, DE) was used as a stationary phase. The PDA data were acquired in the 190–320 nm range and the 200 nm signal was extracted. Gradient elution: mobile phase A (CH_3CN); mobile phase B (H_2O); gradient, 0–2 min, 20% B; 2–15 min, 20%–60% B; 15–16 min, 60% B, 16–18 min, 60%–20% B, 18–21 min, 20% B (column equilibration). Flow rate was 1 mL/min at 25 °C. The experiment was monitored for 24 h. Retention

times were found as follows: NAG-thiazoline **1**, 6.1 min; α-GlcNAc-SH **11a**, 6.8 min; β-GlcNAc-SH **11b**, 7.1 min; oxidation products, 9–13 min (Figure S11).

Figure S11. HPLC Chromatogram of the mixture after NAG-thiazoline decomposition.

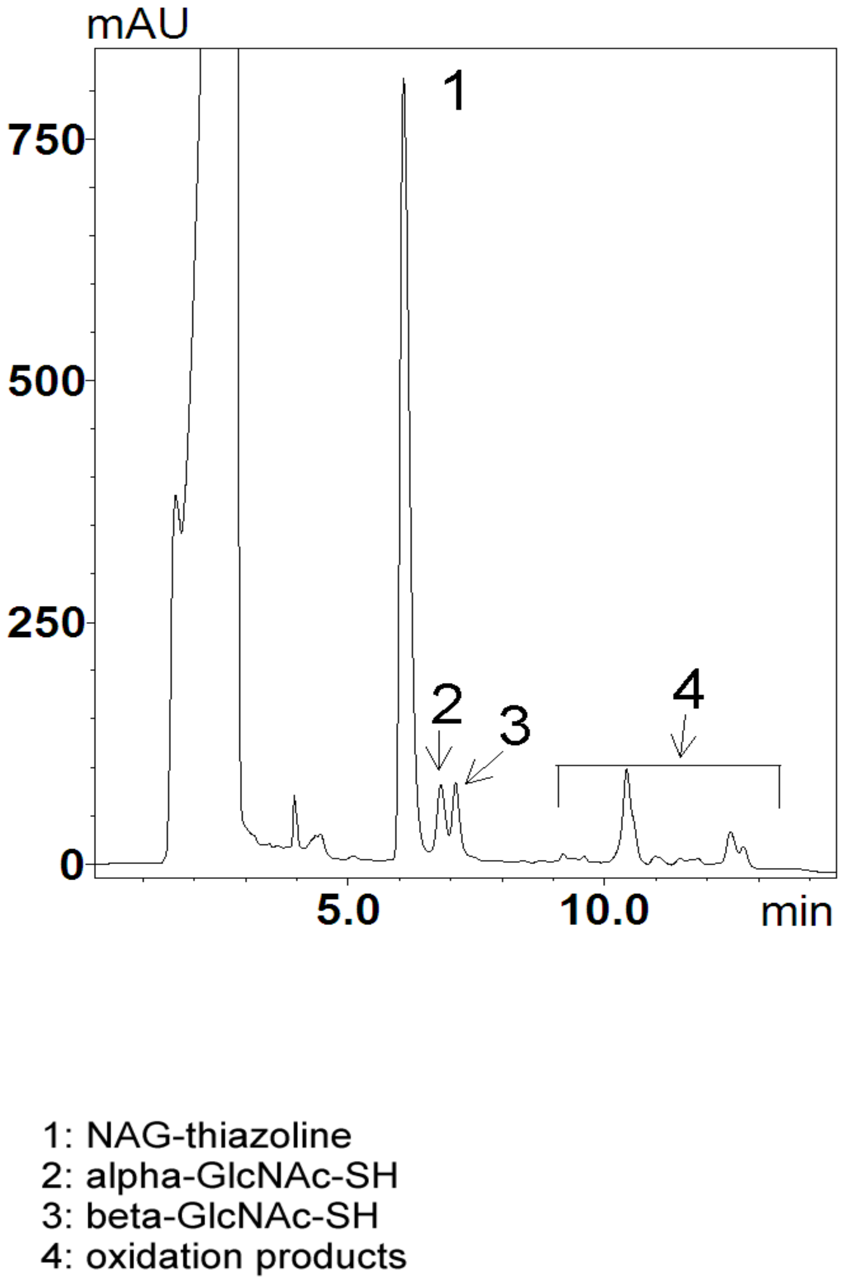

5. HPLC Chromatogram after Reduction of the Mixture of 11a, 11b and 12 by Dithiothreitol

In situ reduction of the products of spontaneous oxidation was performed by adding dithiothreitol (1 M) to the reaction mixture in the final concentration of 100 mM and monitored by HPLC and NMR (Figure S12).

Figure S12. HPLC Chromatogram after reduction of the mixture of **11a**, **11b** and **12** by dithiothreitol.

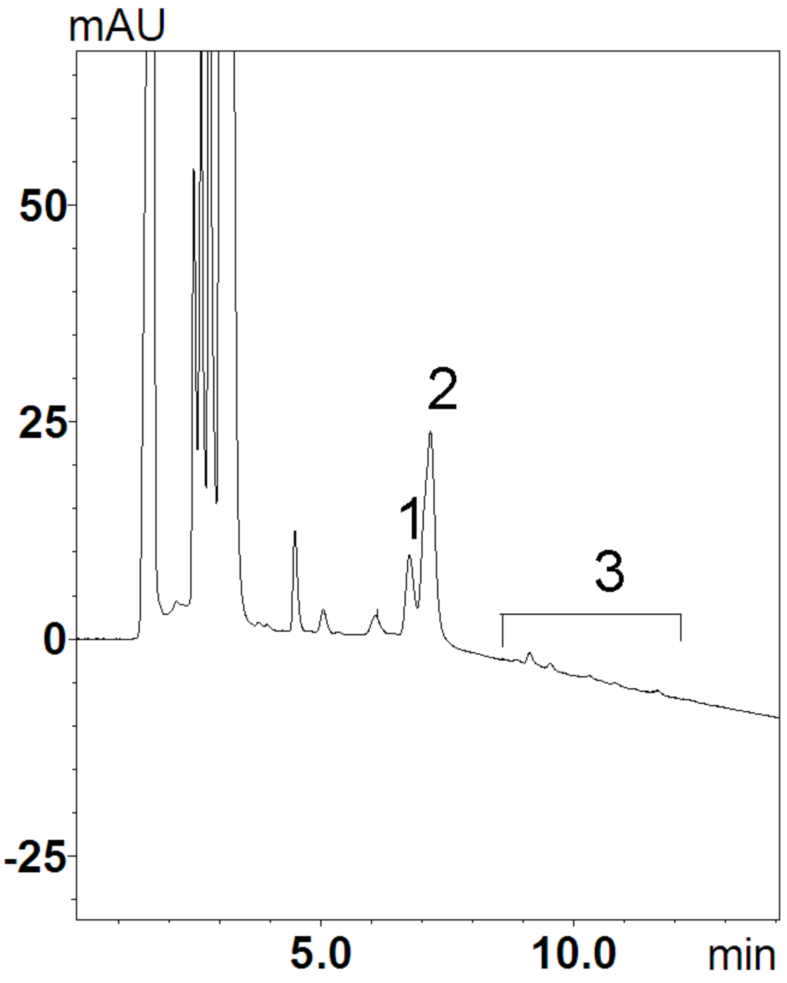

1: alpha-GlcNAc-SH
2: beta-GlcNAc-SH
3: trace oxidation products

β-*N*-Acetylhexosaminidase inhibition by NAG-thiazoline and C-6-azido-NAG-thiazoline. Lineweaver-Burk plots for the individual experiments (Figures S13–S16) are presented.

Figure S13. β-*N*-Acetylhexosaminidase from *Talaromyces flavus* (**A**) NAG-thiazoline, (**B**) C-6-azido-NAG-thiazoline.

A

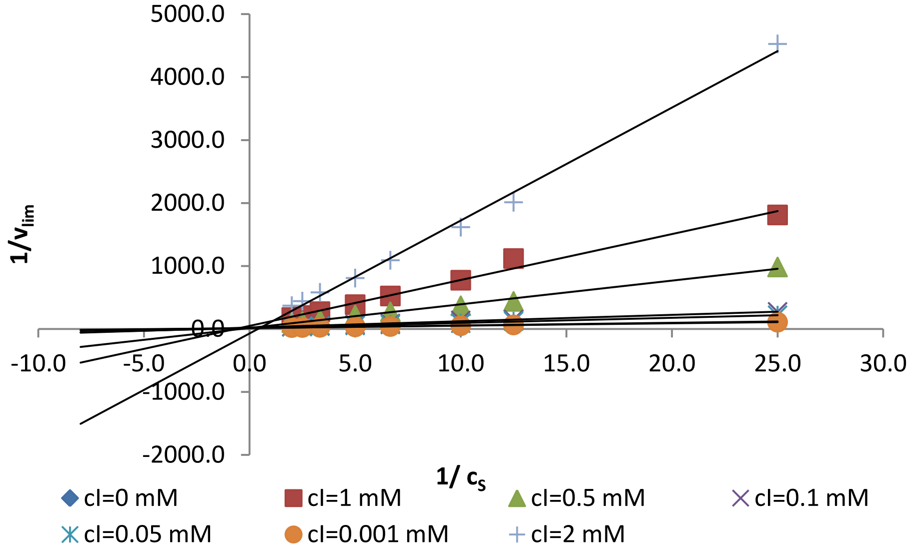

B

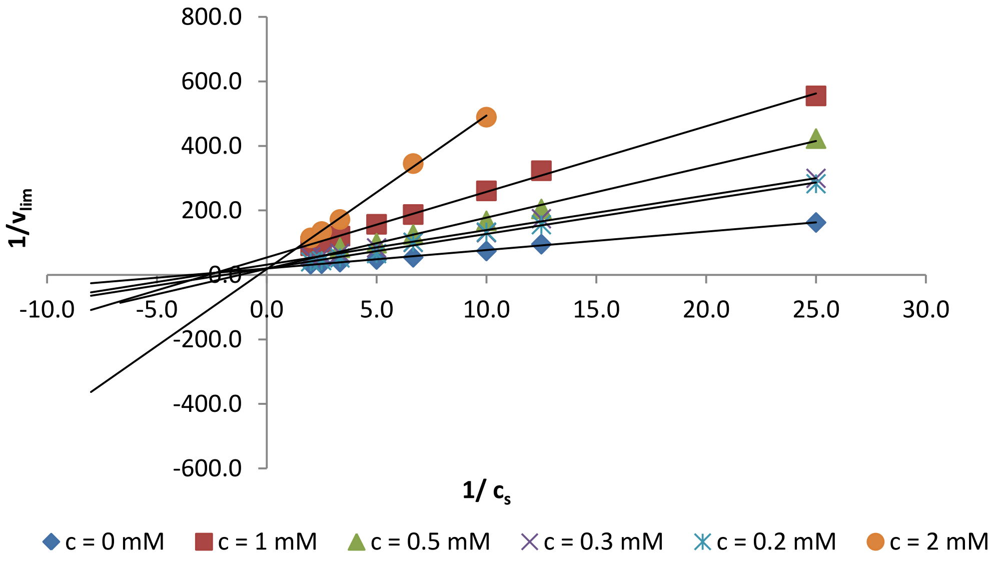

Figure S14. β-*N*-Acetylhexosaminidase from *Streptomyces plicatus* (**A**) NAG-thiazoline, (**B**) C-6-azido-NAG-thiazoline.

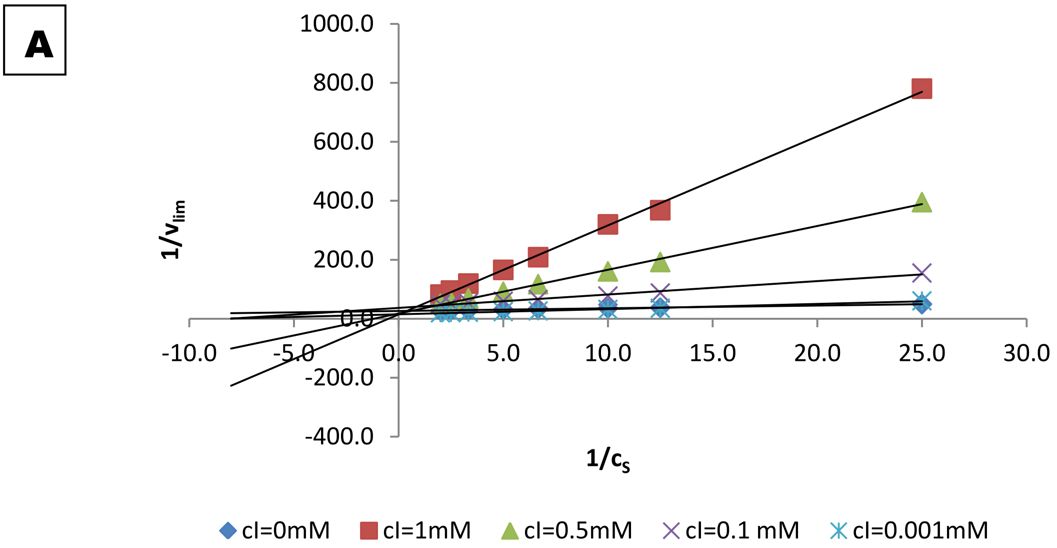

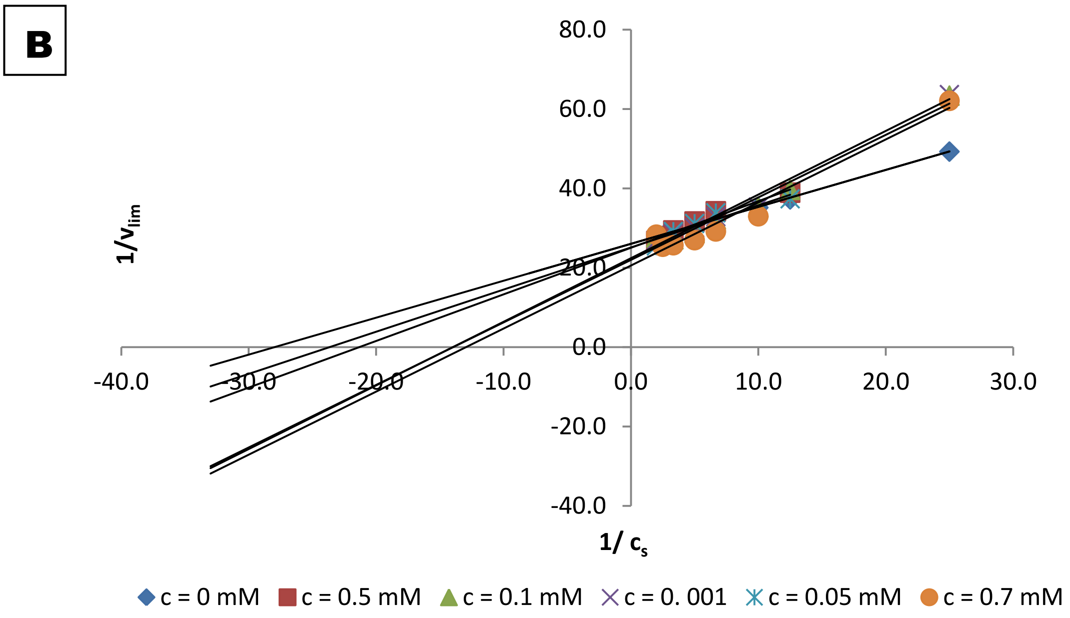

Figure S15. *O*-GlcNAcase from *Bacteroides thetaiotaomicron* (**A**) NAG-thiazoline, (**B**) C-6-azido-NAG-thiazoline.

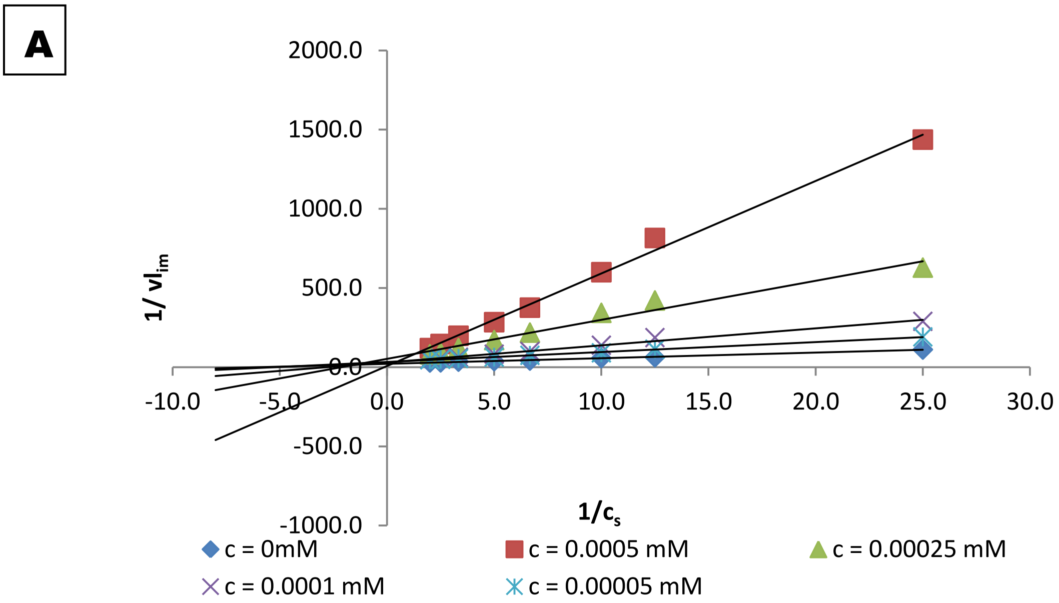

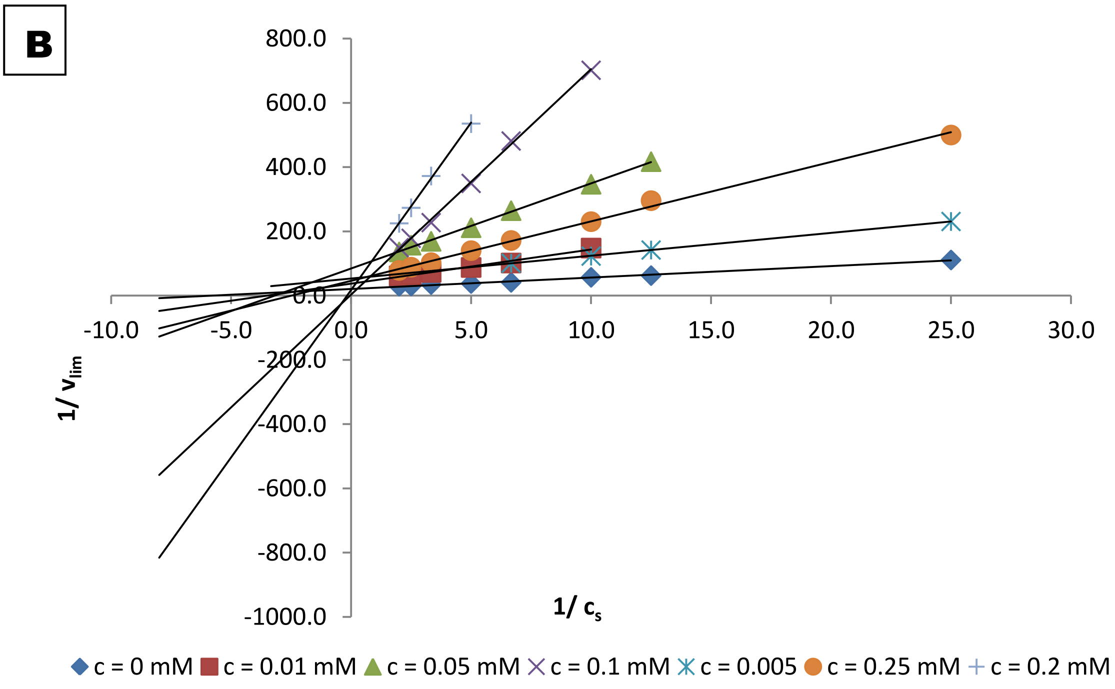

Figure S16. Human *O*-GlcNAcase (**A**) NAG-thiazoline, (**B**) C-6-azido-NAG-thiazoline.

A

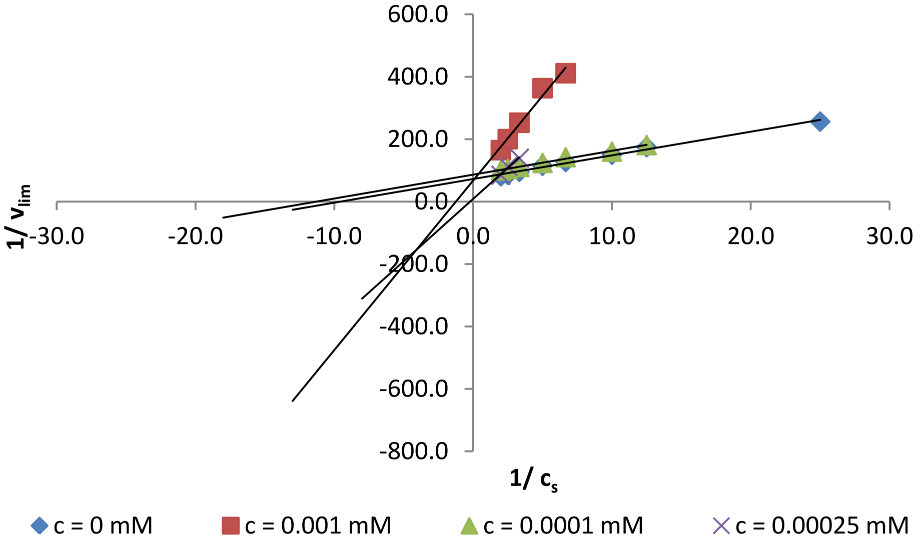

B

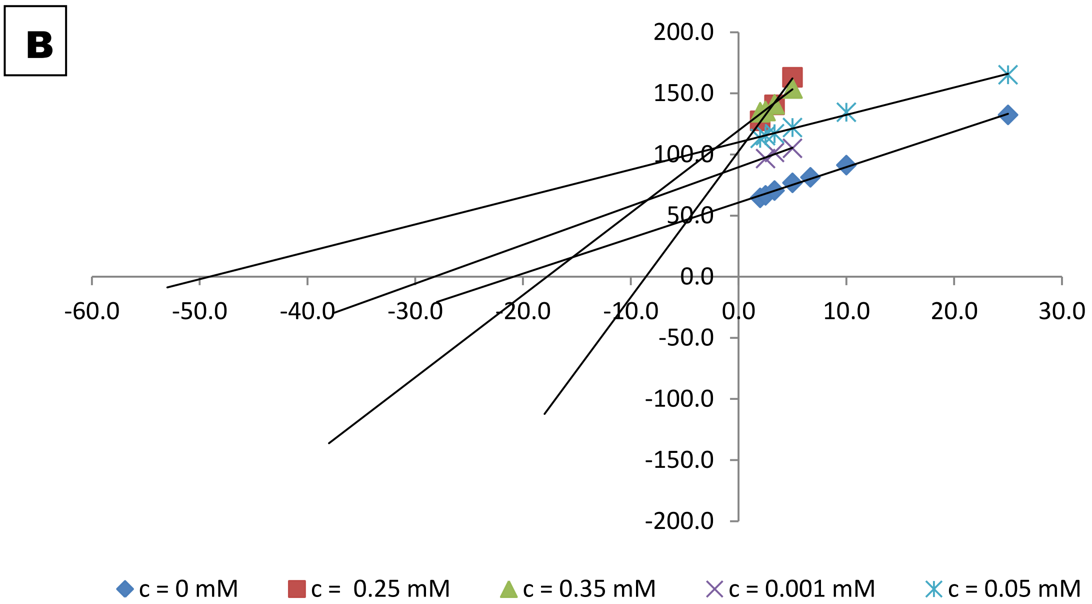

Supplement: Supplementary file 1 [file molecules-19-03471-s001.pdf]
